# Multiphasic blood transcriptomic signatures of radioprotection by BIO 300, a synthetic genistein nanosuspension, in a nonhuman primate model of acute radiation syndrome

**DOI:** 10.1186/s12967-026-08485-4

**Published:** 2026-06-30

**Authors:** Neetha Nanoth Vellichirammal, Stephen Y. Wise, Oluseyi O. Fatanmi, Rachel C. Mingus, Alana D. Carpenter, Sarah A. Petrus, Michael D. Kaytor, Vijay K. Singh

**Affiliations:** 1 Henry M Jackson Foundation for the Advancement of Military Medicine, Bethesda, MD USA; 2 Division of Radioprotectants, Department of Pharmacology and Molecular Therapeutics, F. Edward Hébert School of Medicine, “America’s Medical School”, Uniformed Services University of the Health Sciences, 4301 Jones Bridge Road, Bethesda, MD 20814 USA; 3 Armed Forces Radiobiology Research Institute, Uniformed Services University of the Health Sciences, Bethesda, MD USA; 4 Humanetics Corporation, Minneapolis, MN USA

**Keywords:** BIO 300, Transcriptomics, Temporal change, Acute radiation syndrome, Medical countermeasures, Radioprotection, Genistein, *Macaca mulatta*

## Abstract

**Background:**

Prophylactic radioprotectors for pre-exposure administration are notably absent, creating a critical gap in radiation emergency preparedness and ARS management. BIO 300, a wet-nanomilled formulation of synthetic genistein, is in advanced development as a prophylactic radioprotector with demonstrated survival benefits in murine and nonhuman primate (NHP) models when administered prior to lethal radiation exposure. Longitudinal transcriptomic analysis enables characterization of the molecular mechanisms underlying radioprotective drug action and supports development of potential blood-based monitoring tools for clinical translation. We performed longitudinal blood transcriptome profiling in NHPs receiving 5.8 Gy total-body irradiation to characterize BIO 300‘s radioprotective mechanisms and identify candidate transcriptional biomarkers of drug activity.

**Results:**

BIO 300 demonstrated multiphasic changes in the transcriptome spanning acute cellular preservation (Days 7–10), immune reconstitution (Day 21), and sustained recovery (Day 60), with peak protection spanning 745–865 genes at Days 7–10 and sustained late-phase protection of 558 genes at Day 60. Differential expression analysis revealed four distinct drug-related molecular mechanisms: direct cellular protection, active damage reversal, drug-specific therapeutic responses, and stress attenuation. A core set of 39 genes showing sustained or consistent differential expression was identified, of which 20 carry conventional gene symbols and are functionally interpretable; LOC-designated genes are excluded from functional annotation. Notable annotated genes include SOX2, AKAP11, TIMD4, BTNL10, VNN2, and CLEC1A, representing candidate exploratory transcriptional markers consistent with hematopoietic recovery and immune surveillance. Temporally orchestrated pathway signatures include neuroimmune modulation (Day 7), hemostatic recovery (Day 14), and immunometabolic reconstitution (Day 21).

**Conclusions:**

Our results show that BIO 300 provides multiphasic radioprotection across acute, immune reconstitution, and sustained recovery phases through four distinct mechanisms. Longitudinal transcriptomic signatures identified in this study represent potential blood-based monitoring tools for therapeutic efficacy assessment toward the continued development of BIO 300.

**Supplementary Information:**

The online version contains supplementary material available at 10.1186/s12967-026-08485-4.

## Introduction

Acute radiation syndrome (ARS) is an illness that occurs following partial- or total-body exposure to an acute dose of ionizing radiation delivered at a high dose rate [1, 2]. Severity of the symptoms of ARS are dependent on the radiation dose received and can become lethal when high doses of radiation exposure are reached [3]. ARS manifests in three distinct forms: hematopoietic (H-ARS; 2-6 Gy), gastrointestinal (GI-ARS; 6-10 Gy), and neurovascular (NV-ARS; >10 Gy) subsyndromes [3]. Of these three subsyndromes, NV-ARS is generally considered to be untreatable due to the high doses of radiation that quickly lead to multi-organ failure and death within a few days following exposure [4]. However, drugs are currently being developed for both H-ARS and GI-ARS, as these subsyndromes can still benefit from treatment with radiation medical countermeasures (MCMs) [4]. To date, there are multiple radiation MCMs approved by the United States Food and Drug Administration (US FDA), with four drugs: Neupogen^®^, Neulasta®, Leukine®, and Nplate®, and several biosimilars of Neupogen® and Neulasta® [5–17]. These MCMs are considered radiomitigators, which are administered after exposure to radiation. There are currently no US FDA approved radioprotectors for administration prior to radiation exposure to prevent ARS. This is a critical unmet need, especially in the current global climate, with the potential of radiological and/or nuclear threats. Development of radiation MCMs requires following the guidelines in the US FDA’s Animal Rule, which allows for drugs under development to bypass certain established guidelines when clinical trials with the drug cannot be performed due to ethical limitations [11, 18]. Under these guidelines, the results of well-controlled animal efficacy studies, preferably in two species, can be used in place of human clinical trials. The drug under the development must show safety in animal models, and mechanisms of action must be well-understood. Identification and validation of biomarkers is a critical need during regulatory approval of such MCMs following FDA Animal Rule.

Transcriptomics studies comprehensively characterize genome-wide gene expression changes, providing molecular insights into radiation injury mechanisms and MCM efficacy. Transcriptomic profiling of mRNA and miRNA expression enables identification of biomarkers serving as surrogate endpoints for FDA Animal Rule compliance [19]. These approaches reveal MCM pharmacodynamics, establish dose-response relationships, and identify predictive biomarkers across species [20]. Temporal gene expression patterns may distinguish radioprotectors from radiomitigators, which play an essential role in regulatory approval of countermeasures [21, 22]. Thus, transcriptomics meets the FDA requirements by providing mechanistic understanding and validated biomarkers that are linked to reproducible pharmacodynamic effects [23].

One potential MCM under advanced stages of development is BIO 300, a radioprotectant that can be effective against H-ARS [24]. BIO 300’s active ingredient is synthetically manufactured genistein (5,7-dihydroxy-3-(4-hydroxyphenyl)chromen-4-one), and there are several formulations being developed to allow flexible dosing options [24–27]. Genistein is a soy isoflavone that has demonstrated robust radioprotective efficacy which is mediated via estrogen receptor beta (ERβ) signaling [24–26, 28]. However, poor bioavailability has proven to be an obstacle with genistein’s development as a therapeutic agent and has limited its medical application. To address this, synthetic genistein is wet-nanomilled using a patent protected process resulting in a stable nanoparticle formulation with enhanced bioactivity. Three formulations have been developed: BIO 300 Injectable Suspension (BIO 300 IS), suitable for parenteral administration and currently being evaluated in nonclinical studies; BIO 300 Oral Suspension (BIO 300 OS), suitable for oral self-administration and investigated in both nonclinical and clinical studies [24]; and BIO 300 Oral Powder (BIO 300 OP), an additional oral formulation under clinical investigation [27].

Previous research conducted in the murine and nonhuman primate (NHP) models has established the prophylactic radioprotective efficacy of BIO 300 IS, showing improved survival when given 12–48 h before exposure to a lethal radiation dose [26, 28, 29]. Metabolomic and lipidomic analyses in blood samples have shown transient BIO 300-related changes in amino acid metabolites involved in cellular health and immune functions, and metabolites involved in anti-oxidant activity and fatty acid metabolism [30–32]. Additionally, a phase 1 human safety study of BIO 300 OP investigated transcriptomic changes in the whole blood of individuals dosed orally with BIO 300 OP for multiple days [27]. All formulations of BIO 300 are currently under advanced development as radiation MCMs [24–27].

In this study, the effects of an acute dose of 5.8 Gy total-body radiation exposure on the transcriptomic profile in whole blood were studied and the effect of BIO 300 on the transcriptomic profile of irradiated animals was also investigated. Our results demonstrate that BIO 300 elicits multiphasic changes in gene expression across distinct temporal phases that likely contribute to its prophylactic radioprotective efficacy. We identified a core set of 39 genes that show consistent changes over time. Of these, 20 carry conventional gene symbols and are used for all functional interpretation. Temporally orchestrated pathway signatures including hemostatic recovery (Day 14), neuroimmune modulation (Day 7), and immunometabolic reconstitution (Day 21) provide blood-based monitoring tools with direct clinical translation potential. These findings offer potential blood-based biomarkers for monitoring patients and translating results into clinical use.

## Materials and methods

### Study design and sample collection

**Fig. 1:**
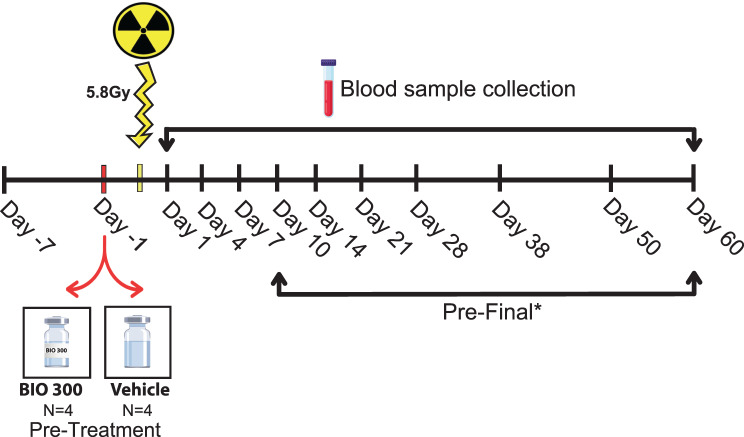
Experimental design for BIO 300 radioprotection study in nonhuman primates. Male NHPs were assigned to either vehicle control (*N* = 4) or BIO 300 IS treatment (*N* = 4) groups during the Pre-Treatment phase. Animals received total-body irradiation (TBI) on Day 0 (indicated by radiation symbol). Blood samples were collected at multiple timepoints: Days −7, 1, 4, 7, 10, 14, 21, 28, 38, 50, and 60 for longitudinal assessment of radioprotection effects and biomarker analysis. Blood samples were also collected before euthanasia at different timepoints (from Days 10–60) defined as Pre-Final

The current study employed a longitudinal time-series design to evaluate the radioprotective effects of BIO 300 compared to the placebo control following exposure to radiation (5.8 Gy). Animals were randomly assigned to either drug treatment or placebo groups and administered the intervention 24 h prior to radiation exposure. Blood samples were collected at multiple times pre- and post-radiation (days −7, 1, 4, 7, 10, 14, 21, 28, 38, 50, 60, and Pre-Final) to assess temporal gene expression changes associated with radiation response and potential drug-mediated radioprotection (Fig. 1). Pre-Final samples were collected before euthanasia at varying timepoints.

#### Animals

This study utilized eight male rhesus macaques (*Macaca mulatta*, Indian sub-strain) as subjects. The animals were mature, ranging in age from 8 to 14 years and weighing between 10.75 and 25.10 kg. These animals were acquired from the National Institutes of Health Animal Center (NIHAC, Poolesville, Maryland). A few years prior to this study, these NHPs served as healthy blood donors. All NHPs were housed individually in a facility that held AAALAC International accreditation. Cage partitions were designed to permit visual and tactile social interaction between animals. A comprehensive environmental enrichment program was implemented to encourage natural behaviors. This included foraging devices and puzzle feeders, mirrors, perches, and rotating manipulation toys. Televisions were provided 3–4 times per week for sensory enrichment. The standard diet consisted of certified primate diet (Purina LabDiet 5048) with continuous access to water. Although precise consumption records were not kept, appetite was closely monitored. A reduction in food intake was specifically noted during the 10–20-day post-irradiation period (the critical period). During this critical time, supplemental items like fruits, vegetables, and oral hydration solutions were administered. Outside of this period, all animals received daily supplements of enrichment foods (including prima treats, peanuts, cereal pieces, yogurt drops, popcorn, marshmallows, etc.) on weekdays. Housing conditions were maintained under a 12-h light/12-h dark cycle, temperature of 22±2 °C, relative humidity of 30–70%, and 10–15 air cycle changes per h. All procedures were conducted in full compliance with the *Guide for the Care and Use of Laboratory Animals* [33] and ARRIVE (Animal Research: Reporting of In Vivo Experiments) guidelines. Additionally, all procedures were approved by the Institutional Animal Care and Use Committee (IACUC) of the Armed Forces Radiobiology Research Institute (AFRRI), Uniformed Services University of the Health Sciences (USUHS), and the Department of War Animal Care and Use Review Office (ACURO).

#### Drug preparation and administration

Before administration, the injection site was prepared following previously established procedures [26]. Briefly, a small area of hair covering the thigh was shaved with a #40 clipper blade and scrubbed at least three times using 4% chlorhexidine and 70% isopropyl alcohol solution. The BIO 300 IS formulation consisted of nanoparticulate genistein (323 mg/mL), 5% povidone K17 (w/w) and 0.2% polysorbate 80 (w/w) in 50 mM phosphate buffered saline (PBS) containing 61 mM sodium chloride [31]. A 23-G, 5/8-inch needle attached to a 3–5 mL syringe was used for all intramuscular (*im)* injections. Each NHP received either a single 50 mg/kg dose of BIO 300 IS or an equivalent volume of vehicle (5% povidone K17 [w/w], 0.2% polysorbate 80 [w/w] in 50 mM PBS with 61 mM sodium chloride) 24 h before TBI [26, 28, 34]. The injection volume ranged from 1.5–3.9 mL and no injection site reaction was observed.

#### Radiation exposure

On the day prior to irradiation, food was withheld (beginning approximately 12–18 h prior to irradiation) to prevent radiation-induced emesis. Animals were sedated 30–45 min before exposure with an *im* injection of a solution containing ketamine and acepromazine at a dose of 10 mg/kg. To ensure minimal movement during irradiation, the anesthetized NHPs were placed in an upright seated position in a restraint box, with limbs secured. Animals were transported individually to the AFRRI high-level cobalt facility (HLCF), where the animal’s identification was verified. Sedation was maintained throughout the exposure using *im* ketamine boosters (0.1–0.3 mL of a 100 mg/mL) as necessary.

The NHPs received a 5.8 Gy total-body ^60^Co γ-radiation at a dose rate of 0.6 Gy/min. The radiation field around the NHP location was uniform within ±1.5%. All NHPs were exposed bilaterally to the abdominal region. This method ensured that each subject received an equivalent radiation dose to the abdominal core regardless of body size. The selected dose of 5.8 Gy corresponds to the LD_30/60_ threshold for rhesus macaques. Dosimetry for the irradiation procedure, specifically regarding dose measurements and calibrations, have been elaborated previously [35, 36]. While undergoing irradiation, animals were continuously monitored by research staff through closed-circuit security cameras. After completing the exposure, the NHPs were transported from the HLCF back to the vivarium and returned to their respective home cages. All animals were monitored by research staff until they were able to stand or sit in an upright position, indicating a recovery from sedation.

#### Blood collection

Blood samples were obtained from sedated animals. Animals were sedated with an *im* injection of a solution containing ketamine and acepromazine at a dose of 10 mg/kg. Peripheral venipuncture was performed on the saphenous vein of the lower leg. The site was disinfected using 70% isopropyl alcohol and dried with sterile gauze immediately before sampling, which served to sterilize the area and improve vein visibility. A 3 mL Luer-lock syringe fitted with a 22–25 G needle was used for blood withdrawal. For transcriptomics, 1 ml of blood was drawn into PAXgene Blood RNA tubes (PreAnalytiX, a Qiagen/Becton, Dickinson and Company, Franklin Lakes, NJ). The blood was mixed immediately by inverting the tube 10 times. The tubes were held at room temperature in the laboratory overnight and were later stored at −80 °C until analysis [37]. Blood samples were collected from a total of twelve timepoints (Day −7, 1, 4, 7, 10, 14, 21, 28, 38, 50, 60, and Pre-Final). Following collection, gentle pressure was applied to the venipuncture site to facilitate hemostasis and prevent the formation of hematoma. The animals were then returned to their home cages and monitored until they were fully recovered from sedation, as well as for potential adverse effects of prolonged bleeding at the collection site.

### Clinical evaluation and supportive care

Following irradiation, animals were monitored a minimum of twice daily for any complications. However, during the critical period (days 10–20 post-irradiation), animals were observed at least three times daily, with 8–10 h between checks. Observations regarding body weight, behavioral changes, or indications of pain/distress were recorded daily. Supportive care was provided based on the presented symptoms of each animal [38]. Supportive care provided included antibiotics (Baytril; enrofloxacin, Bayer HealthCare LLC, Shawnee Mission, KS), fluids, analgesics, antiemetics, and nutritional support. Further details can be found in an earlier publication [39].

### Euthanasia

To avoid unnecessary pain and suffering to the animals, moribundity was used as the surrogate endpoint for mortality. A variety of symptoms were used in determining when an animal reached a state of moribundity. This included, but was not limited to, severe changes to weight or body temperature, minimal responsiveness to stimuli, inappetence, severe anemia or thrombocytopenia, and organ system dysfunction. Moribundity decisions were made only after consulting with a veterinarian. When an animal was deemed moribund, the animal was immediately euthanized. Euthanasia was completed following the *Guide for the Care and Use of Laboratory Animals*, the approved IACUC protocol, and the American Veterinary Medical Association’s (AVMA) Guidelines for Animal Euthanasia [33, 40]. Moribund animals were sedated using ketamine hydrochloride (Mylan Institutional LLC, Rockford, IL) (5–15 mg/kg, *im*), maintained with isoflurane (Baxter Healthcare Corporation, Deerfield, IL) (1–5%) with oxygen at 1–4 liters per minute via mask. Pentobarbital sodium (Virbac AH Inc., Fort Worth, TX) was then administered intravenously or intra-cardiac. Death was confirmed through heart auscultation and the absence of a pulse.

## RNA sequencing and data processing

### Library preparation and sequencing

Total RNA was extracted from peripheral blood samples collected at multiple timepoints pre- and post-irradiation. RNA quality was assessed using Bioanalyzer (Agilent), and samples with RIN scores ≥ 6 were selected for library preparation. RNA-seq libraries were prepared using Illumina HiSeq® X kit and sequenced on Illumina platform generating 150 paired-end reads.

### Read alignment and quantification

Raw sequencing reads were quality-filtered using *FastQC* (v0.11.9) and trimmed to remove possible adapter sequences and nucleotides with poor quality using *Trimmomatic* (v0.36) to remove adapter sequences and low-quality bases [41]. Trimmed reads were aligned to the *Macaca mulatta* reference genome (Mmul_10) using *STAR* aligner (v.2.5.2b) with default parameters. Gene-level read counts were quantified using *featureCounts* from the Subread package (v.1.5.2), counting only uniquely mapped reads overlapping annotated gene features. The hit counts were summarized and reported using the *gene_id* feature in the annotation file. Only unique reads that fell within exon regions were counted.

### Data normalization

Raw count matrices were imported into R (v4.3.1) for downstream analysis. Low-abundance genes were filtered, retaining only genes with >10 reads in at least 5 samples. Additional filtering removed genes with zero variance and those exhibiting extreme outlier ratios (maximum count/median count i.e., outlier ratio > 100) to ensure robust statistical analysis. Library sizes were assessed across all samples, and principal component analysis (PCA) was performed on variance-stabilized transformed (VST) counts to evaluate sample clustering, treatment separation, and temporal trajectories. Library size normalization and variance stabilizing transformation were performed using *DESeq2* (v 1.42.1) to account for technical variation across samples.

### Differential gene expression analysis

Gene expression analysis was performed using DESeq2 (version 1.42.1) to identify genes differentially expressed between treatment groups at each timepoint [42]. Genes were first filtered to retain only those with at least 10 counts in a minimum of 5 samples, non-zero variance across samples, and an outlier ratio (maximum count/median count) ≤100.

### Time-point specific analysis using standard approach

A DESeqDataSet object was created using a grouped design formula (design = ~group), where “group” represents the combination of treatment condition and timepoint (e.g., Drug_Day7, Vehicle_Day7). The DESeq2 workflow was executed with default parameters, which includes estimation of size factors using the median-of-ratios method, dispersion estimation with shrinkage toward the fitted trend, and negative binomial generalized linear model (GLM) fitting. For each timepoint, pairwise comparisons between drug-treated and vehicle control samples were performed with the contrast parameter specifying Drug vs. Vehicle groups. Differentially expressed genes (DEGs) were identified using a false discovery rate (FDR) threshold of 0.05 (Benjamini-Hochberg adjustment) and an absolute log2 fold change threshold of ≥1.0 (corresponding to a minimum 2-fold change).

## Temporal pattern analysis

### Temporal phase classification

To facilitate interpretation of transcriptional changes across the experimental timeline, samples were classified into four temporal phases based on days post-irradiation and the known biology of ARS in NHPs: Pre-radiation (baseline, Day −7), Early response (Days 1–4), Peak response (Days 7–21), and Recovery (Days 28–60). The Pre-radiation phase represents a single pre-irradiation blood collection at Day −7, which served as the baseline reference for within-group temporal comparisons. The Early response (Days 1-4) captured the immediate post-irradiation window prior to onset of overt hematopoietic injury. The Peak phase (Days 7–21) corresponded to the period of maximum hematopoietic suppression and peak immune dysregulation in the NHP H-ARS model, consistent with previously published clinical and transcriptomic trajectories in irradiated rhesus macaques [37]. The Recovery phase (Days 28–60) spanned the extended follow-up period during which hematopoietic reconstitution and transcriptional normalization were expected. Pre-Final samples, collected at variable timepoints prior to euthanasia, were analyzed separately and not assigned to a phase due to variable timing across animals. This classification scheme enabled systematic assessment of treatment-induced transcriptional dynamics across distinct biological response windows.

Temporal transcriptomic changes were also analyzed separately within each treatment group using *DESeq2*, with Day 1 as the reference timepoint (design formula: ~ timepoint). This captures radiation-induced transcriptional changes in vehicle-treated animals and the combined radiation and drug response in BIO 300-treated animals over time. Comparing these two trajectories identifies timepoints where BIO 300 diverges from the radiation-only response, complementing the primary cross-sectional Drug vs. Vehicle comparisons described above. For each subsequent timepoint (Days 4, 7, 10, 14, 21, 28, 38, 50, 60, and Pre-Final (moribund animal, just prior to terminal procedure), DEGs were identified using thresholds of adjusted *p*-value < 0.05 (Benjamini-Hochberg correction) and |log2 fold change| ≥1. Pre-Final samples, collected immediately prior to euthanasia of moribund animals at variable timepoints between Days 10 and 60, were included for two reasons: first, they capture the transcriptional state associated with ARS severity and radiation-induced mortality, providing biologically informative data on the most severely affected animals; second, they allow assessment of whether BIO 300-associated transcriptional patterns persist to the terminal stage in treated animals. Because Pre-Final samples are not collected at a fixed time point and the number of contributing animals is variable and limited, all Pre-Final findings are interpreted descriptively and are not used to draw quantitative conclusions about temporal trajectories. Pre-Final data are reported in supplementary tables and displayed separately in all figures. Genes were then classified into five mutually exclusive pattern categories based on their responses in drug versus vehicle groups at each timepoint: (1) Protected - genes showing significant changes in vehicle but not in drug-treated animals (vehicle: P_adj_ < 0.05, |log2FC| ≥1; drug: P_adj_  ≥ 0.05 or |log2FC| <1), indicating preservation of baseline expression; (2) Reversed - genes changing significantly in both groups but in opposite directions (vehicle and drug: P_adj_ < 0.05, |log2FC| ≥1, opposite signs); (3) Drug Specific - genes changing significantly only in drug-treated animals (drug: P_adj_ < 0.05, |log2FC| ≥1; vehicle: P_adj_ ≥ 0.05 or |log2FC| <1), representing drug-induced transcriptional responses; (4) Attenuated - genes changing significantly in both groups in the same direction, but with drug response magnitude less than 50% of vehicle response, indicating partial protection; and (5) Unprotected - genes changing significantly in both groups in the same direction with drug response magnitude ≥ 50% of vehicle response, indicating the drug provides less than 50% reduction in radiation-induced changes. Pattern assignments were performed independently at each timepoint to capture temporal dynamics of drug-mediated radioprotection.

### Time-series pattern Classification

To identify genes exhibiting consistent temporal expression patterns in response to treatment, we applied a pattern classification algorithm based on the frequency, direction, and magnitude of differential expression across timepoints. For each gene, we calculated (1) The number of timepoints showing significant differential expression (adjusted *p*-value < 0.05), (2) The number of timepoints with significant upregulation (log2FC ≥ 1), (3) The number of timepoints with significant downregulation (log2FC ≤ −1and (4) Maximum absolute fold change across all timepoints.

Genes were classified into temporal patterns as follows: *Sustained Up*- Genes significantly upregulated (log2FC ≥ 1, adjusted *p* < 0.05) at ≥ 5 timepoints, *Sustained Down*- Genes significantly downregulated (log2FC ≤ −1, adjusted *p* < 0.05) at ≥ 5 timepoints, *Consistent Up*- Genes significantly upregulated (log2FC ≥ 1, adjusted *p* < 0.05) at ≥ 3 timepoints, *Consistent Down*- Genes significantly downregulated (log2FC ≤ −1, adjusted *p* < 0.05) at ≥ 3 timepoints, *Mixed*- Genes showing significant changes (adjusted *p* < 0.05) at > 2 timepoints with both upregulation (log2FC ≥ 1) and downregulation (log2FC ≤ −1) observed, and *Transient*- Genes showing significant changes (adjusted *p* < 0.05) at 1–2 timepoints only. Genes not meeting significance criteria at any time point were classified as *Not Significant*.

### Terminal time point analysis

For genes classified as showing sustained or consistent temporal patterns, we evaluated whether these expression changes persisted to the terminal sacrifice timepoint (Pre-Final). A gene was considered to “persist to terminal” if it exhibited the same directional change (log2FC ≥ 1 for upregulated patterns or log2FC ≤ −1 for downregulated patterns) with statistical significance (adjusted *p* < 0.05) at the terminal timepoint.

### Pathway and functional enrichment analysis methods

Comprehensive functional enrichment analysis was performed using the *clusterProfiler* (v4.10.1), *ReactomePA *(v1.46.0), and *STRINGdb* (v2.14.3) R packages on DEGs identified across experimental timepoints. Due to limited pathway annotation for *Macaca mulatta* (Rhesus Macaque, Mmul_10 genome), macaque gene symbols were first converted to human orthologs via Ensembl BioMart (v2.58.2, using the mmulatta_gene_ensembl dataset), filtering for one-to-one orthologous relationships to ensure mapping reliability. Human orthologs were subsequently converted to Entrez Gene IDs using the org.Hs.eg.db (v3.18.0) annotation database. DEGs were defined using thresholds of adjusted *p*-value (P_adj_) <0.05 and absolute log2 fold change ≥ 1.0.

Gene Ontology (GO) enrichment was performed separately for all DEGs, upregulated DEGs, and downregulated DEGs using the enrichGO function in the *clusterProfiler* package with ontology=“BP” (Biological Process), organism database org.Hs.eg.db, and Benjamini-Hochberg multiple testing correction (pAdjustMethod=“BH”). KEGG pathway enrichment was conducted using the enrichKEGG function in the *clusterProfiler* package with organism=“hsa” (human) and identical statistical parameters. Reactome pathway enrichment was performed using the enrichPathway function from ReactomePA package with organism=“human”, enabling readable gene names. For all three enrichment methods, significance thresholds were set at P_adj_ value < 0.05, with gene set size boundaries of minimum 10 and maximum 500 genes per term/pathway to exclude overly specific or broad functional categories. At timepoints with very few DEGs, enriched terms may achieve statistical significance (P_adj_ < 0.05) while being supported by only a single DEG (Count = 1). Such terms are reported in supplementary tables but are explicitly flagged in the Results as non-conclusive, as single-gene support does not constitute evidence of coordinated pathway-level activity regardless of adjusted *p*-value.

### Visualization and data presentation

PCA trajectories were visualized using the first two principal components, with temporal progression indicated by connecting samples from the same treatment group in chronological order. Time-point specific PCA plots were generated using faceted layouts to assess treatment separation at each measurement time.

Summary statistics for each temporal pattern included: gene count per pattern category, percentage of analyzed genes, mean and maximum fold changes, and the proportion of time points showing significant differential expression. Pattern assignments were validated through visual inspection of gene-level temporal expression profiles.

## Results

### Overview of study design and sample characteristics

We conducted a time-series RNA-seq study to evaluate the radioprotective effects of BIO 300 in NHPs exposed to ionizing radiation. Animals received either drug or vehicle 24 h prior to radiation exposure, with blood samples collected at multiple timepoints post-irradiation for transcriptomic profiling. The average RIN (RNA Integration Number) was >9 confirming high RNA quality across samples. RNA-seq libraries generated a mean sequencing depth of 32.6 million reads per sample (range: 21.1–44.7 million). Total read alignment rate to the *Macaca mulatta* reference genome (Mmul_10) was 94.5% (range: 58.1–98.7%), with a mean unique mapping rate of 90.2% (range: 56.7–95.6%). Five samples showed mapping rates below 80%, attributable to low RNA input quantity rather than RNA degradation, as confirmed by RIN values of 6.1–10.0 for these samples. After quality filtering, we obtained expression data for 19,553 genes across 77 samples representing 12 timepoints in both treatment groups. The number of biological samples contributing to each timepoint varied due to animal deaths. A complete breakdown of samples per group per timepoint is provided in STable 1. Briefly, both groups contributed *n* = 4 samples at most scheduled timepoints through Day 21; sample availability in the vehicle group declined at later timepoints due to radiation-induced mortality, with surviving animals contributing samples through Day 60. Pre-Final samples represent collections immediately before euthanasia of moribund animals and vary in number and timing across animals. Unless otherwise specified, all differential expression analyses compare BIO 300-treated animals with vehicle-treated animals at the corresponding time point.

### Principal component analysis and sample clustering

Principal component analysis (PCA) revealed distinct clustering patterns between drug-treated and vehicle control samples across the study timeline (Suppl Figure 1, 2). PCA was performed on VST-normalized counts from all samples and visualized to assess treatment group separation (BIO 300 vs. vehicle) and temporal structure. The first two principal components explained 42.1% of the total variance (PC1: 27.4%, PC2: 14.7%). Drug-treated samples showed slight separation from vehicle controls along PC1, with the magnitude of separation varying across different phases of the study (Pre, Early, Peak, and Recovery phases). The temporal trajectory analysis demonstrated progressive changes in gene expression profiles over time, with samples colored by days post-treatment revealing a continuous shift in transcriptional state from baseline through the 60-day period.

**Fig. 2:**
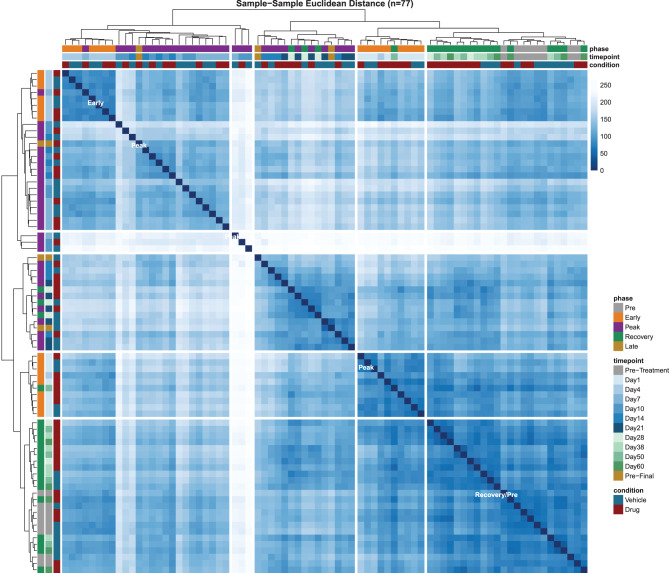
Hierarchical clustering heatmap showing Euclidean distances between all sample pairs (*n* = 77) based on whole blood transcriptome data. Color intensity represents pairwise distance, with darker colors indicating greater transcriptomic similarity and lighter colors indicating greater dissimilarity. Samples are annotated by experimental phase (Baseline: Day −7; Early response: Days 1–4; Peak response: Days 7–21; recovery: Days 28–60; Pre-Final: variable terminal timepoint), individual animal ID, timepoint (Pre-Treatment through Day 60 and Pre-Final), and treatment condition (BIO 300 vs. vehicle). Five major clusters, broadly corresponding to the temporal phases, are outlined by boxes on the heatmap. Cluster boundaries are approximate and reflect dominant phase-level grouping; within-cluster intermixing between treatment conditions reflects the moderate rather than complete treatment-driven separation observed across the study timeline

Hierarchical clustering using Ward’s method showed partial grouping of drug-treated (red circles) and vehicle control (blue circles) samples, with notable intermixing between treatment groups (Suppl Figure 3). Treatment conditions influence sample clustering to some extent, but it is neither the sole nor dominant factor driving clear separation. Temporal phases (indicated by colored squares) contribute additional structure, though samples from different timepoints and treatments remain intermixed

**Fig. 3:**
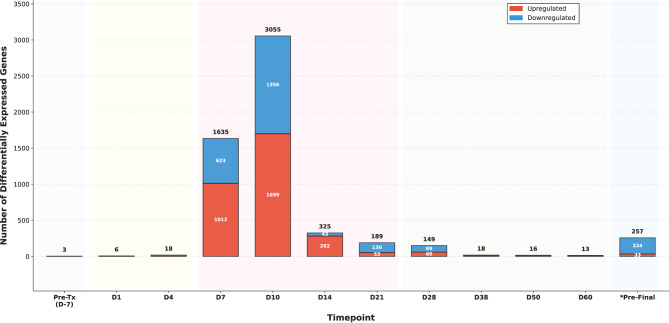
Temporal dynamics of BIO 300-mediated transcriptomic response reveal distinct phases of radioprotection. Stacked bar chart showing the number of differentially expressed genes (DEGs) between BIO 300-treated and vehicle control animals at each timepoint (Padj < 0.05, |log2FC| ≥1.0). Red bars represent genes upregulated in BIO 300-treated relative to vehicle-treated animals; blue bars represent downregulated genes. Total DEG counts are labeled above each bar, with directional counts shown within bars. Background shading indicates temporal phases: baseline (Day −7), early response (Days 1–4), peak response (Days 7–21), and recovery (Days 28–60). The Pre-Final bar (asterisk) represents samples collected immediately prior to euthanasia at variable timepoints between Days 10 and 60. Analysis is based on 77 blood samples across 12 timepoints. Red star indicates that samples defined as Pre-Final were collected before euthanasia at variable timepoints (from Days 10–60)

The sample-to-sample Euclidean distance heatmap similarly demonstrates moderate within-group similarity. Substantial overlap between treatment groups was identified, with many drug-treated samples showing intermediate distances to vehicle controls rather than clear between-group divergence (Fig. 2). Five major clusters are visible along the diagonal of the heatmap, broadly corresponding to the Pre, Early, Peak, Recovery and Late phases; these clusters are highlighted in Figure 2 to aid interpretation. The dominant clustering of samples by temporal phase rather than treatment condition in the global transcriptomic structure (Fig. 2) indicates that BIO 300‘s effects represent a modulation of the shared radiation response rather than a wholly distinct transcriptional program, and all treatment-specific findings reported here should be interpreted within this context.

Collectively, these results indicate that drug treatment induces measurable changes in the transcriptional landscape across the experimental timeline, but the treatment effect is moderate rather than producing complete and distinct separation from vehicle controls.

### Temporal patterns of differential gene expression

All time point-specific DEG counts reflect pairwise comparisons of BIO 300-treated vs. vehicle-treated animals at each respective timepoint, using FDR < 0.05 and |log2FC| ≥1. Differential expression analysis across the 12-timepoint longitudinal study identified dynamic transcriptional responses to drug treatment. At Pre-Treatment (Day −7), only 3 DEGs were detected between BIO 300 and vehicle-treated animals (Drug vs. Vehicle, Day −7), representing pre-existing baseline differences between groups prior to any intervention. The Early response phase (Days 1–4) showed minimal differential gene expression, with only 6 and 18 DEGs detected at Day 1 and Day 4, respectively (Table 1, Fig. 3). This reflects shared radiation responses in both groups rather than indicating an absence of transcriptional activity. To examine the underlying response more directly, within-group analyses comparing Day −7 baseline to Days 1 and 4 were performed separately in vehicle-treated and BIO 300-treated animals. Vehicle-treated animals showed 5,729 DEGs on Day 1 (2,965 upregulated, 2,764 downregulated) and 1,941 DEGs at Day 4 (691 upregulated, 1,250 downregulated) relative to Pre-Treatment baseline (FDR < 0.05, |log2FC| ≥1; STable 2A, B). BIO 300-treated animals showed a comparable early response, with 4,398 DEGs at Day 1 (2,230 upregulated, 2,168 downregulated) and 1,837 DEGs at Day 4 (835 upregulated, 1,002 downregulated; STable 3A, B). A substantial fraction of these genes was shared between groups, including 3,634 genes at Day 1 (63.4% of vehicle Day 1 DEGs) and 1,116 genes at Day 4 (57.5%), indicating that much of the early post-irradiation transcriptional response was common to both groups. Because the Drug vs. Vehicle comparisons (at specific days after irradiation) are designed to detect treatment-specific differences, these shared radiation-induced changes are not expected to appear strongly in that contrast. Overall, the minimal DEG counts at Days 1 and 4 likely reflect biological convergence of early radiation responses between groups, consistent with prior reports of early transcriptional responses after irradiation in rhesus macaques [37]. A dramatic transcriptional shift occurred on Day 7, with 1,635 total DEGs (1,012 upregulated, 623 downregulated), in BIO 300-treated relative to vehicle-treated animals, and this robust differential expression was sustained through Day 10 (3,055 DEGs) before gradually declining through the remainder of the Peak response phase. By Day 14, differential expression had substantially diminished to 325 DEGs and reached minimal levels during the Recovery phase at Days 38–60 (13–18 DEGs).

**Table 1:** Differentially expressed genes in drug-treated and control animals at different timepoints. Differentially expressed genes (DEGs) were identified using a false discovery rate (FDR) threshold of 0.05 (Benjamini-Hochberg adjustment) and an absolute log2 Fold change threshold of ≥1.0

Time point	Days	Genes Upregulated	Genes Downregulated	Total Genes Differentially Expressed
Pre-Treatment	−7	3	0	3
Day1	1	3	3	6
Day4	4	17	1	18
Day7	7	1012	623	1635
Day10	10	1699	1356	3055
Day14	14	282	43	325
Day21	21	53	136	189
Day28	28	60	89	149
Day38	38	14	4	18
Day50	50	13	3	16
Day60	60	11	2	13
Pre-Final*		33	224	257

*Pre-Final samples were collected immediately prior to euthanasia at variable timepoints between Days 10 and 60; no single day value is assigned

### Identification of sustained and consistent transcriptional responses

**Fig. 4:**
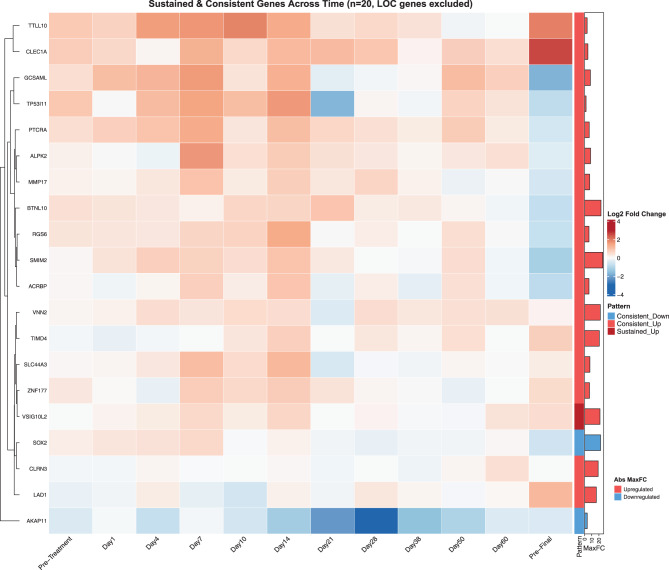
Sustained and consistent differential expression patterns across 20 priority genes. Heatmap showing Log2 Fold Change values (Drug vs Vehicle) for the 20 annotated priority genes with sustained or consistent differential expression across the post-irradiation time course. Pattern categories are defined by frequency of significant differential expression (P_adj_ < 0.05, |log₂FC| ≥1): Consistent Up (red) and Consistent Down (dark blue)—significant at ≥ 3 timepoints; Sustained Up (dark red), significant at ≥ 5 timepoints. These 20 genes are the annotated subset of the full 39-gene set (STable 6); the remaining 19 carry LOC designations are excluded from functional annotation. The MaxFC sidebar indicates the absolute maximum fold change magnitude for each gene, with bars colored red for upregulated patterns and blue for downregulated patterns. Timepoints span Pre-Treatment through Day 60 across experimental phases (Early, Peak, Recovery). Red star indicates that samples defined as Pre-Final were collected before euthanasia at variable timepoints (from Days 10–60)

Pattern classification was applied to differentially expressed genes (BIO 300 vs. vehicle) across all longitudinal timepoints. Because both groups received identical radiation exposure, genes responding to radiation alone with equivalent changes in both groups are not detected in this analysis. Consequently, all sustained and consistent patterns described in this section represent BIO 300-specific transcriptional responses. Among genes showing differential expression between BIO 300-treated and vehicle-treated animals at one or more timepoints, pattern classification identified two groups of interest for biological interpretation. The first comprised genes with transient gene expression (22.4%, significant at 1–2 time points, STable 4). In total, 39 genes met criteria for sustained or consistent differential expression (STable 4); of these, 20 carry conventional gene symbols and are used for all functional interpretation below (Fig. 4), while the remaining 19 LOC-designated genes of unknown function are listed in STable 4. Among these priority genes, 17 showed consistent upregulation (Consistent Up pattern), 2 displayed consistent downregulation (Consistent Down pattern), and 1 exhibited sustained upregulation (Sustained Up pattern) (STable 4). The Consistent Down genes showed the highest median maximum fold change (18.47) and were significant across an average of > 4 timepoints (STable 5). Consistent Up genes displayed a mean maximum fold change of 12.20 (median: 7.38) across an average of > 3 timepoints, while Sustained Up genes showed a mean maximum fold change of 13.96 (median: 14.88) and were significant across an average of > 6 timepoints, demonstrating the most persistent differential expression pattern (STable 6).

**Fig. 5:**
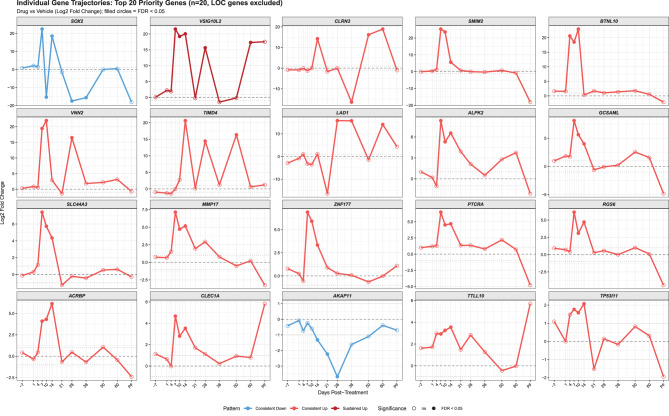
Temporal expression trajectories of top 20 priority genes. Faceted line plots showing Log2 Fold Change (drug vs vehicle) over time for 20 annotated priority genes meeting sustained or consistent differential expression criteria (see methods). Consistent Up/Down: gene is significantly differentially expressed (P_adj_ < 0.05, |log₂FC| ≥1) in the same direction at ≥ 3 timepoints. Sustained Up: gene meets significance criteria at ≥ 5 timepoints with consistent upregulation and is assigned preferentially to this category over consistent Up. Filled circles indicate time points reaching statistical significance (FDR < 0.05, |log₂FC| ≥1); open circles indicate non-significant timepoints. Y-axis scales are set independently per gene panel to maximize dynamic range visibility. Genes are displayed in order of decreasing number of significant time points within each pattern category. Pre-Final (PF) samples were collected at variable timepoints prior to euthanasia and are not fixed longitudinal timepoints

Notable genes within the consistent upregulation pattern included immune-regulatory molecules (CLEC1A, TIMD4, BTNL10), which play roles in dendritic cell function, T-cell regulation, and antigen presentation, as well as VNN2, a pantetheinase enzyme involved in both immune cell trafficking and vitamin B5 metabolism (STable 6). The sustained upregulation pattern included genes with the highest number of significant timepoints (mean: 6.25 timepoints), demonstrating the most persistent elevation across the study duration with a mean maximum fold change of 13.96 (STable 6). Among the consistently downregulated genes were SOX2 (max FC: 22.38, significant at 5 timepoints), and AKAP11 (max FC: 3.68, STable 6). Individual temporal profiles of the top 20 priority genes, ranked by number of significant timepoints and maximum fold change, are shown in Fig. 5. Consistent directional separation between BIO 300-treated and vehicle control samples was observed across the post-irradiation time course.

### Pathway enrichment analysis

Pathway enrichment was performed on DEGs identified from BIO 300 vs. vehicle comparisons at each timepoint using three complementary databases (GO Biological Process, KEGG, Reactome) revealed convergent temporal patterns of drug-mediated radioprotection (STable 7–36). GO Biological Process provided broad coverage of immune and cellular processes; Reactome contributed mechanistic resolution of reaction-level pathways including coagulation cascades and phagocytic signaling; and KEGG uniquely resolved metabolic pathway organization and provided the primary evidence for GABAergic synapse enrichment underlying the neuroimmune modulation signature at Day 38. The three databases were therefore non-redundant in their biological coverage across the temporal response. Day 21 consistently emerged as the timepoint with maximum biological complexity across all three methods: 141 GO biological processes, 40 KEGG pathways, and 20 Reactome pathways, representing peak immunometabolic integration. Days 50 and 60 showed differentially expressed genes but no coordinated pathway-level enrichment, with Reactome returning only single-gene terms that do not reflect biological programs (STable 24–25).

Early Response Phase (Days 4–10): Day 4 showed limited differential expression (18 DEGs total), and pathway enrichment results at this timepoint should be interpreted with caution, as each enriched GO term was driven by a single gene. With this caveat, 12 GO terms (P_adj_ < 0.05) touched on oxygen/gas transport, ion homeostasis, and early immune activation (STable 7). Similarly, KEGG and Reactome returned terms related to cornified envelope formation and neutrophil degranulation (STables 17, 27); however, these reflect single-gene hits within large gene sets and do not indicate coordinated pathway-level activity. These findings are reported for completeness but do not support biological conclusions at this timepoint. Day 7 exhibited robust and interpretable functional enrichment with 31 GO terms (adjusted *p* < 0.05) spanning neurological processes (glial cell migration, P_adj_ = 0.001), cellular communication (synapse organization), tissue morphogenesis, ion homeostasis, and sensory perception, indicating complex multi-system biological reprogramming consistent with the onset of peak hematopoietic injury (STable 8). Notably, Days 7 and 10 showed no Reactome pathway enrichment despite substantial DEG counts, demonstrating that pathway-level coordination, rather than gene abundance, defines critical biological transitions. Day 10 GO enrichment focused exclusively on collagen metabolic process (21 genes mapped from input DEG list, STable 9).

Day 14 demonstrated pronounced hemostatic restoration across all three databases. GO enrichment returned 11 terms dominated by coagulation processes, including hemostasis (P_adj_ = 2.87E-06), blood coagulation (P_adj_ = 8E-06), platelet activation (P_adj_ = 0.0009), and wound healing (STable 10). KEGG identified platelet activation as the sole significant pathway (8 genes, P_adj_ = 0.006; STable 30). Reactome returned two significant terms: platelet activation, signaling and aggregation (P_adj_ = 0.005, *n* = 12) and formation of fibrin clot (P_adj_ = 0.009, *n* = 5; STable 20). Together, these findings indicate coordinated coagulation cascade reactivation following radiation-induced thrombocytopenia.

Day 21 represented the peak of biological complexity across all three databases. GO enrichment (141 terms) spanned adaptive immunity (leukocyte mediated immunity, lymphocyte activation, P_adj_ < 0.001), T-cell biology (activation, proliferation, differentiation), antigen processing and presentation (MHC class II protein complex assembly P_adj_ < 0.001), myeloid cell functions, immune regulation, cytokine signaling, and metabolic processes (Suppl Figure 4, STable 11). KEGG analysis (40 pathways) identified 18 immunity-related pathways (antigen processing, B/Fcγ receptor signaling, phagocytosis, leukocyte migration), 7 pathogen defense pathways (tuberculosis, leishmaniasis, influenza A), 6 cellular architecture pathways (actin regulation, focal adhesion, tight junctions), and 7 metabolic pathways (steroid biosynthesis, glycolysis/gluconeogenesis, STable 31). Reactome analysis (20 pathways) specifically highlighted innate immune activation (phagocytic cup formation, platelet/neutrophil degranulation P_adj_ = 0.005–0.01), Fcγ receptor-dependent phagocytosis (P_adj_ = 0.01), MHC class II antigen presentation (P_adj_ = 0.03), pathogen defense (Leishmania phagocytosis), metabolic reprogramming (gluconeogenesis, GLUT4 translocation), and cytokine signaling (JAK-STAT after IL-12 stimulation, STable 21). This convergent evidence across databases identifies Day 21 as a transcriptomic inflection point characterized by signatures consistent with the onset of immune reconstitution and restoration of multi-system homeostasis.

Late Recovery Phase (Days 28–60): Day 28 showed cell cycle-focused enrichment (GO: 6 terms including chromosome segregation, mitotic nuclear division (STable 12); Reactome: M Phase, Mitotic Prometaphase, STable 22), indicating proliferative activity for tissue remodeling in BIO 300-treated relative to vehicle-treated animals. Day 38 demonstrated neuroimmune modulation primarily through KEGG and Reactome databases. However, as with Day 4, GO enrichment results at Day 38 should be interpreted with caution: all 25 GO terms with P_adj_ < 0.05 were driven by single-gene hits (STable 13) and do not reflect coordinated pathway-level activity. KEGG analysis identified 6 pathways focused on amino acid and neurotransmitter metabolism (GABAergic synapse, alanine/aspartate/glutamate metabolism, STable 33). Reactome identified GABA synthesis/degradation and neurotransmitter release cycle (STable 23), collectively providing the primary evidence for neuromodulatory signaling at this time point, though all single gene hits. The GO terms and Reactome data are reported for completeness but do not independently support biological conclusions.

At Day 50, no significant GO-BP enrichment was detected at the adjusted *p*-value threshold (P_adj_ < 0.05; STable 14). Reactome returned 9 terms passing Padj < 0.05 (STable 24); however, all 9 were single-gene hits and do not reflect coordinated pathway-level activity. These findings are reported for completeness but do not support biological conclusions at this timepoint. At Day 60, no significant GO-BP or KEGG enrichment was detected at the adjusted *p*-value threshold (P_adj_ < 0.05; STable 15, STable 35). Reactome returned 2 nominally significant terms (STable 25), both driven by single-gene hits, and do not support coordinated pathway-level conclusions at this timepoint.

Collectively, this multi-database approach reveals a triphasic recovery pattern: hemostatic crisis resolution by Day 14, transcriptomic signatures consistent with the onset of immune reconstitution at Day 21, and cell cycle-focused tissue remodeling at Day 28. A neuroimmune transcriptomic signal was detected at Day 7, supported by multi-gene GO enrichment; nominal pathway enrichment at Day 38 was driven entirely by single-gene hits and is noted for completeness, without implying coordinated pathway-level activity or central nervous system involvement.

### BIO 300 exhibits biphasic radioprotection through preservation of cellular homeostasis

In this section, “protected genes” refers to genes that changed significantly in irradiated vehicle-treated animals but stayed near baseline in BIO 300-treated animals at the same time point, i.e., BIO 300 prevented the irradiation-associated change in their expression. BIO 300 demonstrates temporally dynamic radioprotective effects with biphasic kinetics. Protection peaked during the acute phase at Days 7–10 (745–865 protected genes), where irradiation-associated transcriptional changes observed in vehicle-treated animals were absent in BIO 300-treated animals (STable 37–38). Time-course patterns were determined by comparing each group’s expression at each post-irradiation timepoint to Day 1 within that group separately; pattern classifications apply to post-irradiation timepoints only (Days 4–60 and Pre-Final). Within-group analyses confirmed that both vehicle and BIO 300 animals showed robust irradiation-associated transcriptional responses at Days 1 and 4 relative to Pre-Treatment baseline (5,729 and 4,398 DEGs respectively), with 3,634 shared genes at Day 1 (63.4% overlap), confirming that the protected gene patterns at Days 7–10 emerge against a background of shared early irradiation responses in both groups (STable 2A&B, STable 3A&B). Following minimal Day 4 response (7 genes), protection rapidly increased by Day 7 (745 genes). After the acute peak, protection exhibited a complex temporal pattern: decline to Day 14 (422 genes), secondary elevation at Day 21 (681 genes), reaching a minimum at Day 38 (131 genes), then substantial late-phase resurgence at Day 60 (558 genes) and Pre-Final (436 genes). This biphasic pattern reflects protection during both acute injury and late-phase recovery. Across all post-irradiation timepoints, 2,638 unique genes were classified as protected at one or more timepoints (non-redundant union of 4,111 cumulative gene-timepoint instances; STable 37), indicating broad but temporally dynamic transcriptome preservation.

The drug employed four distinct protective mechanisms across timepoints: (1) Direct protection - genes changing significantly in vehicle animals but remaining stable in BIO 300-treated animals (745–865 genes at Days 7–10); (2) Active reversal - genes changing significantly in both groups but in opposite directions, indicating active transcriptional opposition rather than simple stabilization most prominent at Day 7 (141 genes) and Day 10 (98 genes), including immune regulators CD74, B2M, CSF1R, and TYROBP (STable 37); (3) Drug-specific responses - transcriptional programs activated uniquely in BIO 300-treated animals with no corresponding change in vehicle animals, strongest at Days 7–10 (~2,800 genes) including interferon-stimulated genes such as IFIT1; and (4) Attenuated responses - genes changing significantly in both groups in the same direction but with a reduced magnitude in BIO 300-treated animals (<50% of vehicle response magnitude), representing partial protection rather than complete stabilization (33 unique genes, concentrated at Days 7–10; STable 37–38). Across all post-irradiation timepoints, the four mechanisms involved 2,638 protected, 252 reversed, 6,214 drug-specific, and 33 attenuated genes (unique gene counts, STable 37–38).

**Fig. 6:**
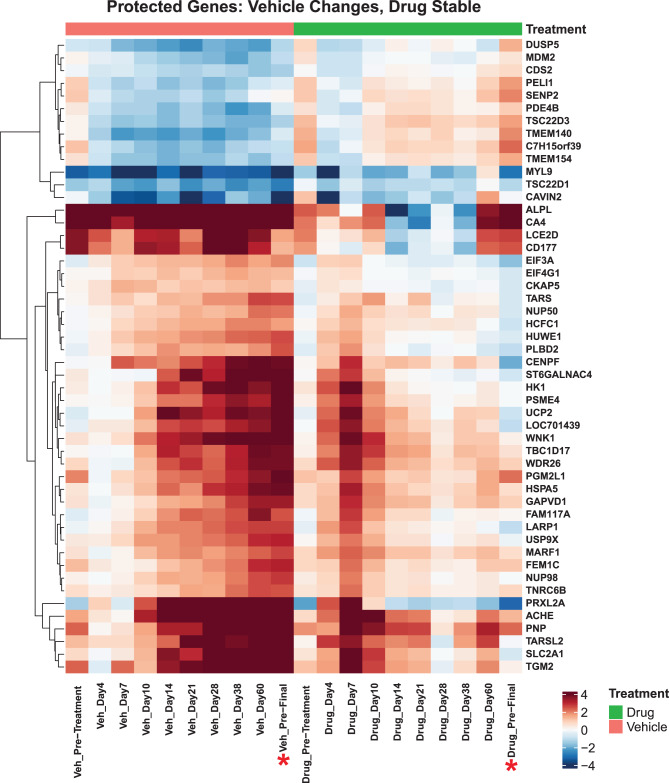
BIO 300 protects against radiation-induced transcriptomic changes in key cellular pathways. Heatmap of 50 representative protected genes showing differential temporal expression patterns between vehicle and drug (BIO 300) treatment groups. Protected genes show significant radiation-induced expression changes in vehicle-treated animals (P_adj_ < 0.05, |log₂FC| ≥1, within-group vs. Day 1) that are absent in BIO 300-treated animals at the same timepoint, indicating preservation of baseline expression. These 50 genes were selected from 2,638 unique protected genes (STable 38) to represent key biological pathways discussed in the results. These genes were prioritized by maximum Fold change and breadth of temporal protection. Color scale represents log2 normalized expression from −4 (blue, low) to + 4 (red, high) across timepoints from Pre-treatment through Pre-Final. Pre-Final samples (indicated by asterisk) were collected before euthanasia at variable timepoints and are not fixed longitudinal timepoints

Protected genes revealed coordinated preservation of critical cellular systems with temporal progression (Fig. 6). Early protection (Days 7–10) included two distinct patterns. First, stress response regulators were markedly downregulated in the vehicle-treated animals but remained stable with BIO 300, including DUSP5 (a MAPK phosphatase that provides negative feedback on ERK signaling), glucocorticoid-responsive transcriptional regulators TSC22D1 and TSC22D3. Although these genes are classically associated with stress-induced upregulation, their downregulation in vehicle-treated animals may reflect radiation-induced exhaustion of negative feedback regulation or suppression of glucocorticoid-dependent anti-inflammatory signaling; BIO 300 preserves their baseline expression, maintaining normal regulatory tone. Second, translational machinery components EIF3A, EIF4G1, and TARS were upregulated in vehicle-treated animals while remaining stable in BIO 300-treated animals, suggesting BIO 300 prevents radiation-induced translational stress responses. DNA damage response and cell cycle regulators began showing protection at Day 10 including MDM2 (a key p53 regulator), CENPF (a centromere-associated mitotic protein), nuclear pore complex component NUP50, and translation factor LARP1 (all upregulated in vehicle, but stable in BIO 300). Because MDM2 negatively regulates p53, its downregulation in vehicle-treated animals indicates elevated p53 activity consistent with radiation-induced DNA damage; preservation of MDM2 in BIO 300-treated animals suggests attenuation of excessive p53-mediated apoptotic signaling rather than absence of p53 involvement. From Day 14, protection expanded to include additional systems: ER stress response (HSPA5), metabolic homeostasis genes SLC2A1 (glucose transporter GLUT1), HK1 (hexokinase), and TGM2 (tissue transglutaminase), plus additional nuclear transport components (NUP98), all showing significant upregulation in vehicle-treated animals that were absent from BIO 300-treated animals. Beyond direct protection, active reversal patterns were also observed at Day 7, with immune-associated genes including CD74, B2M, CSF1R, and TYROBP showing significant changes in opposite directions between groups, suggesting possible modulation of radiation-associated immune responses. Additionally, drug-specific transcriptional responses at Days 7–10 (~2,800 genes), including interferon-stimulated genes such as IFIT1, were detected exclusively in BIO 300-treated animals, with no corresponding changes in vehicle controls.

Late-phase protection at Day 60 (558 genes) exceeded Day 28 (266 genes), demonstrating sustained multi-system radioprotection beyond acute injury. Most protected genes persisted including CENPF, NUP50, NUP98, HSPA5, DUSP5, TSC22D1, TSC22D3, SLC2A1, HK1, TGM2, and the translation machinery. Notably, MDM2 protection was limited to Days 10–28, potentially reflecting completion of DNA damage response resolution by Day 60. This sustained protection of metabolic regulators, translation factors, and stress response genes suggests maintained cellular homeostasis during tissue remodeling and repair phases (STable 36).

## Discussion

This comprehensive longitudinal transcriptomic analysis in a clinically relevant NHP model demonstrates that blood-based gene expression profiling provides sensitive and dynamic detection of both radiation injury and BIO 300-mediated radioprotection. Findings from this study provide molecular evidence for BIO 300‘s sustained systemic protection and identify key biological processes underlying its radioprotective efficacy under conditions relevant to the FDA Animal Rule requirements for countermeasure development.

### Blood transcriptome as a systemic biomarker of radiation response and drug-mediated rescue

This study demonstrates that peripheral blood transcriptomics serves as a powerful systemic biomarker for monitoring total-body responses to radiation injury and therapeutic intervention with BIO 300, a genistein-based radioprotective countermeasure. Blood-based gene expression analysis has emerged as a realistic approach for assessing radiation exposure due to its high throughput capacity and time efficiency, with demonstrated use in biodosimetry applications ranging from low-dose medical exposures to ARS [43, 44]. This longitudinal time-series design revealed that blood transcriptome signatures capture integrated systemic responses across multiple organ systems, as shown by the detection of coordinated pathway activation spanning hemostatic, immunological, metabolic, and neuroimmune domains.

A recent genome-wide blood transcriptomic study identified 34 previously undescribed radiation-responsive genes with dose-dependent expression, reinforcing that the blood transcriptome captures a rich and dose-sensitive radiation signal [45]; the BIO 300 drug-specific transcriptional responses identified here emerge against this shared radiation background, highlighting the analytical challenge and biological specificity of detecting drug effects in a radiation injury context.

### BIO 300-mediated radioprotection: mechanism and context

BIO 300, a pharmaceutically optimized nanosuspension of synthetic genistein, represents a promising radiation MCM currently under advanced development for both H-ARS and the delayed effects of acute radiation exposure (DEARE) [31, 46]. Genistein has demonstrated radioprotective efficacy through multiple mechanisms including selective agonistic activity for estrogen receptor beta (ERβ), attenuation of radiation-induced proinflammatory markers (IL-1β, IL-6, COX-2), and promotion of hematopoietic recovery [30].

Previous NHP studies have confirmed BIO 300’s safety and efficacy when administered prophylactically (24 h pre-irradiation). In the pilot study with younger rhesus macaques (ages 2.5–7 years, 5.8 Gy TBI), BIO 300 achieved 100% survival versus 50% in controls. A subsequent study in older adults (ages 8–14 years) demonstrated 75% versus 50% survival, validating efficacy across age groups [26, 29]. Critically, BIO 300-treated animals exhibited superior hematological recovery with higher nadirs and faster post-nadir recovery for neutrophils, platelets, erythrocytes, hemoglobin, and hematocrit. The most pronounced improvements occurred in erythrocyte parameters, with significantly higher hemoglobin on days 21 and 24 post-irradiation. Additionally, BIO 300-treated animals showed reduced hemorrhage, bone marrow atrophy, and GALT depletion [47]. Our transcriptomic analysis extends these findings by revealing the temporal molecular mechanisms underlying BIO 300’s protective effects.

### Pattern-specific mechanisms of BIO 300 radioprotection

BIO 300’s radioprotective effects exhibit a multiphasic temporal pattern with three distinct protective peaks. During the acute injury phase (Days 7–10) when both GI and hematopoietic damage are fully expressed [48, 49], BIO 300 achieves maximum protective capacity, maintaining baseline expression patterns across 745–865 genes. The peak of BIO 300-mediated protection at Days 7 to 10 coincides with the period of maximal gastrointestinal mucosal injury, a phase now recognized to involve distinct cell death mechanisms beyond classical apoptosis. Recent work identified ferroptosis (an iron-dependent, oxidative cell death pathway) as the primary mode of radiation-induced depletion of oral mucosal epithelial cells, with fibroblast-derived polyamines providing a critical early compensatory metabolic shield [50]. The preservation of metabolic homeostasis genes observed in BIO 300-treated animals at Days 7 to 10, including the glucose transporter SLC2A1 (GLUT1) and hexokinase HK1, may reflect a complementary mechanism by which BIO 300 supports the energetic requirements of mucosal cell survival under ferroptotic stress. Consistent with this, restoring zinc homeostasis in irradiated oral mucosa via a zinc tannate nanoparticle adhesive patch that activates the KLF5/FoxO signaling axis in mucosal fibroblasts substantially prevents radiation-induced mucosal ulceration [51]. This further supports the emerging concept that metabolic and micronutrient support of stromal-epithelial crosstalk is a therapeutically tractable mechanism in radiation mucosal injury. Whether BIO 300’s systemic transcriptomic protection extends to preservation of comparable fibroblast-epithelial metabolic support circuits in the gastrointestinal mucosa warrants investigation in tissue-specific follow-up studies. Protection remains substantial at Day 21 (681 genes), coinciding with the period of maximum immune reconstitution. Late-phase sustained protection resurges at Day 60 (558 genes), demonstrating sustained efficacy against DEARE [52].

Pathway enrichment revealed mechanistically distinct processes across four protection patterns. Protected genes (2,638 unique genes across post-irradiation timepoints; STable 38) showed sustained enrichment for proteasome-mediated protein degradation and p53 signaling. MDM2, a key negative regulator of p53, was downregulated in vehicle-treated animals but maintained at baseline levels in BIO 300-treated animals, suggesting that BIO 300 preserves normal p53 checkpoint regulation rather than allowing radiation-induced dysregulation of this pathway. HSPA5 similarly reflects preserved proteostasis essential for cellular survival [53, 54]. Reversed genes (252 unique genes; peak 141 at Day 7), including immune regulators such as CD74, B2M, CSF1R, and TYROBP, were enriched for immune response pathways including antigen presentation (MHC class I and class II) and antigen processing. CD74 participates in MHC class II assembly and trafficking, while B2M is essential for MHC class I complex formation. CSF1R and TYROBP support antigen-presenting cell function through macrophage differentiation and immune cell signaling, respectively. This coordinated upregulation is consistent with restoration of antigen presentation capacity and modulation of radiation‑induced immunosuppression [55, 56]. Drug-specific genes (6,214 unique genes; 2,684 at Day 7 and 2,872 at Day 10), including interferon-stimulated IFIT1, were enriched for immune response activation and pathways related to tetrapyrrole/iron metabolism and myeloid differentiation, processes that support erythropoiesis and host defense after radiation injury [57, 58]. However, because the study design did not include a non-irradiated BIO 300-treated control group, it cannot be determined whether these drug-specific transcriptional programs reflect responses to radiation-induced injury specifically modulated by BIO 300, or whether they partly represent baseline pharmacological activity of genistein, including its known estrogenic and immunomodulatory effects that would be present regardless of radiation exposure. Interpretation of the drug-specific gene category should therefore remain cautious, and these findings are best considered as candidate radioprotective signatures requiring validation in a study design that includes a drug-only, non-irradiated arm. Attenuated genes (33 unique genes, predominantly at Days 7–10; STable 37–38) showed late enrichment for UV protection and translational regulation, indicating partial rather than complete protection of stress-adaptive responses during the acute injury phase [59].

This multi-modal mechanism, simultaneously activating protective pathways, preventing catastrophic stress responses, and reversing immunosuppression, fundamentally distinguishes BIO 300 from single target radioprotectors such as amifostine [60]. By maintaining cellular and immunological homeostasis across the acute-to-recovery transition, BIO 300 offers a broader spectrum of radioprotection.

### Molecular architecture of radioprotection: 20 priority genes orchestrate stem cell differentiation and immune surveillance

A core set of 39 genes (1.5% of 2,582 analyzed) exhibited sustained or consistent differential expression between BIO 300-treated and vehicle groups: 32 consistently upregulated, 3 consistently downregulated, and 4 sustained upregulation (mean > 6 timepoints, mean maximum FC 13.96). Of these 39 genes, 19 carry LOC designations and lack conventional functional annotation. After excluding these, 20 annotated genes remain, comprising 17 Consistent Up, 2 Consistent Down, and 1 Sustained Up. All functional interpretations below refer to these 20 annotated genes. The complete 39-gene set is provided in STable 4. These represent candidate transcriptional markers of genistein-mediated radioprotection, reflecting molecular mechanisms conferring survival advantage.

BIO 300-treated animals showed consistent upregulation of immune-regulatory molecules: TIMD4, a phosphatidylserine receptor mediating apoptotic cell clearance and T-cell regulation [61]; BTNL10, involved in T-cell co-stimulation and immune checkpoint modulation [62]; VNN2 (GPI-80), a pantetheinase involved in inflammation, oxidative stress, and leukocyte migration [63, 64] and CLEC1A, modulating dendritic cell function during inflammation [65]. This coordinated upregulation is consistent with transcriptional programs associated with immune surveillance and radiation-damaged cell clearance, though whether these gene expression changes translate to functional immune activity cannot be established from blood RNA-seq data alone and requires cellular and functional validation.

SOX2 showed the strongest downregulation in BIO 300-treated animals (maximum FC = 22.38, 5 timepoints), suggesting a shift in stemness-associated transcriptional programs. SOX2 maintains stem cell self-renewal and pluripotency [66] so its suppression may reflect BIO 300-induced altered HSPC state recovery, rather than direct accelerated differentiation. AKAP11, which anchors and regulates PKA signaling, was similarly downregulated [67]. Together, these changes are consistent with BIO 300 modulating transcriptional programs linked to hematopoietic recovery, although the functional consequences will require further validation. This interpretation is compatible with prior reports that genistein, the active ingredient of BIO 300 promotes HSPC quiescence prior to radiation exposure [48]. These data suggests that radioprotective effects of BIO 300 may involve phase-specific regulation of HSPC activity, protection through quiescence during irradiation, followed by transcriptional changes consistent with support hematopoietic recovery rather than a simple switch from quiescence to activation.

Sustained upregulation patterns (5–8 timepoints) represent prolonged radioprotective mechanisms: enhanced hematopoietic reserve, immune surveillance, and tissue repair. These 20 genes, demonstrating consistent differential expression across multiple independent timepoints in BIO 300-treated animals relative to vehicle-treated animals, represent candidate exploratory biomarkers of genistein-mediated radioprotection at the transcriptional level. Their temporal stability is a necessary but not sufficient criterion for pharmacodynamic utility; protein-level quantification, functional validation, and RT-PCR confirmation in independent cohorts will be required before these candidates can be considered validated pharmacodynamic endpoints. Their identification here is therefore reported as hypothesis-generating, providing a prioritized set of targets for future mechanistic and translational studies.

### Temporal dynamics of transcriptional response identifies Day 21 as the critical inflection point

BIO 300’s radioprotective effects manifest through distinct biological phases. Days 7–10 represent the acute protective period when BIO 300 maximally preserves baseline cellular functions, preventing radiation-induced transcriptional dysregulation across 745–865 genes during peak GI and hematopoietic injury. Day 21 emerges as the critical reconstitution phase, when immune and metabolic systems achieve maximum coordinated functional recovery, marking the transition from acute protection to systemic regeneration.

Pathway enrichment analysis revealed 20 Reactome pathways, and 141 GO biological processes significantly enriched at this timepoint, representing the most extensive activation across the entire study timeline. This timepoint involves adaptive and innate immunity (antigen processing/presentation, B/Fcγ gamma receptor signaling, phagocytosis), pathogen defense preparedness (tuberculosis, leishmaniasis, influenza A responses), cellular architecture remodeling (actin cytoskeleton, focal adhesions, tight junctions), and metabolic reprogramming (gluconeogenesis, glycolytic process, pyruvate metabolic process). This comprehensive activation pattern demonstrates that Day 21 represents the critical window during which BIO 300-mediated drug rescue promotes immune reconstitution and the restoration of multi-system homeostasis.

The temporal dynamics observed in our study align with established principles of immune reconstitution following hematopoietic injury. Radiation-induced hematopoietic stem cell (HSC) injury causes acute depletion and long-term functional impairment. Residual radiation damage manifests as decreased HSC reserves, impaired self-renewal, and defective lymphocyte reconstitution. Studies in irradiated mice have shown that immune reconstitution follows distinct temporal patterns, with myeloid recovery preceding lymphoid restoration. Complete reconstitution of lymphoid tissues, including the thymus and spleen, will require extended periods [68]. Our findings at Day 21 aligns temporally with the expected window of robust immune cell regeneration and functional restoration following successful hematopoietic rescue [69].

The Day 21 inflection point identified in our transcriptomic analysis corresponds precisely with the timeframe when BIO 300-mediated hematopoietic recovery is evident. BIO 300-treated animals showed significantly higher red blood cell (RBC) counts (*p* < 0.05) with similar patterns for hemoglobin and hematocrit at this timepoint [47]. This temporal convergence between transcriptional reprogramming and functional recovery demonstrates that blood transcriptome directly reflects the biological processes driving BIO 300’s therapeutic efficacy.

### Platelet activation signatures in radiation-induced hemostatic crisis resolution

The focused hemostatic pathway activation at Day 14, characterized by platelet activation (P_adj_  < 0.01) and coagulation cascade engagement, reflects critical recovery from radiation-induced thrombocytopenia. Radiation exposure causes severe thrombocytopenia through multiple mechanisms including direct megakaryocyte damage, disruption of the bone marrow vascular niche, and release of negative paracrine factors such as platelet factor 4 (PF4) that inhibit megakaryopoiesis [43, 70]. Thrombocytopenia represents a significant cause of morbidity and mortality after radiation exposure, with platelet counts correlating strongly with survival after TBI [71]. The megakaryocyte lineage exhibits a particular vulnerability to radiation, with recovery requiring coordinated regeneration of hematopoietic progenitors, megakaryocyte differentiation, and restoration of platelet production capacity [72].

Our transcriptomic data showing platelet activation signatures at Day 14 directly correlates with the hematological findings from the NHP survival studies. BIO 300-treated animals demonstrated improved platelet nadirs and exhibited significantly higher platelet levels on day 24 (*p* < 0.05), with shorter duration to return to baseline compared to vehicle-treated animals [47]. Importantly, while all decedent animals and one vehicle-treated survivor exhibited moderate thrombocytopenia (<20,000 cells/µL), BIO 300-treated animals showed faster recovery trajectories. This suggests that the Day 14 hemostatic pathway activation detected in blood transcriptome represents an early molecular signature of accelerated megakaryopoiesis and platelet regeneration, preceding the measurable improvements in circulating platelet counts observed at later timepoints.

Previous studies have demonstrated that recombinant thrombopoietin and growth factors can promote hematopoietic reconstitution and enhance megakaryocyte generation following radiation injury [73]. The detection of these pathways in peripheral blood indicates that circulating immune cells and platelets reflect the systemic restoration of hemostatic capacity, providing a non-invasive window into bone marrow regenerative processes.

### Neuroimmune modulation in the radiation response

Beyond hematopoietic and immune reconstitution, our transcriptomic analysis revealed activation of neuroimmune pathways in peripheral blood. This is consistent with the ability of circulating immune cells, especially monocytes, T cells, and granulocytes to express neurotransmitter receptors and produce neuroactive molecules. These cells can also respond to neuroimmune signals outside the central nervous system [74, 75]. This suggests systemic radiation responses engage neuroimmune regulatory circuits detectable in blood. Prominent neuroimmune signatures were observed at Days 7 and 38, characterized by neurotransmitter metabolism pathways (GABA synthesis, glutamate metabolism) and neurological GO terms (glial cell differentiation, synapse organization). On Day 7, 31 GO terms included neurological processes (glial cell migration- P_adj_  < 0.01, telencephalon migration- P_adj_ = 0.03). On Day 38 nominal enrichment of GABA synthesis, release, reuptake and degradation (P_adj_  = 0.024, Reactome; STable 22) and amino acid neurotransmitter metabolism pathways (KEGG; STable 32) were detected; however, all terms at this timepoint were driven by single-gene hits and do not reflect coordinated pathway-level activity. As this dataset is derived entirely from peripheral whole-blood bulk RNA-seq, these findings reflect transcriptional changes in circulating immune cells and cannot be directly interpreted as evidence of central nervous system protection or neuroimmune-axis remodeling. Whether BIO 300 influences neuroimmune signaling beyond the peripheral compartment requires tissue-specific investigation.

Emerging evidence demonstrates extensive bidirectional communication between the nervous and immune systems, with neurotransmitters serving as critical immunomodulatory molecules [76]. GABA, traditionally recognized as the primary inhibitory neurotransmitter in the central nervous system, is produced by peripheral immune cells including T cells and macrophages, where it functions as a negative regulator of inflammatory cytokine production and T cell activation [74]. This peripheral GABAergic signaling is transcriptionally detectable in blood and modulates cytokine secretion, proliferation, migration, and cytotoxicity, with implications for autoimmune diseases and inflammatory responses. Glutamate similarly exhibits dual functions in neurotransmission and immune regulation, with altered glutamate-GABA balance contributing to neuroinflammation in peripheral immune compartments [76–78].

The detection of these neuroimmune pathways in peripheral blood transcriptome suggests that radiation exposure and therapeutic intervention engage central-peripheral neuroimmune circuits. The central nervous system regulates peripheral immune responses through autonomic nervous system outputs, with specific neural circuits transferring information about peripheral inflammation and transmitting regulatory signals via sympathetic and parasympathetic pathways [79]. Radiation is known to affect the nervous system, inducing changes in neurotransmitter receptor expression, synaptic plasticity, and neuroinflammation [80]. Our findings suggest that the successful radiation countermeasure mechanism of BIO 300 may operate partly through modulation of neuroimmune interactions, representing an underappreciated component of systemic radioprotection.

## Clinical implications and future directions

The temporal transcriptomic architecture revealed in this study has several clinical implications. First, the identification of Day 21 as the critical inflection point for immune reconstitution suggests this timepoint represents an optimal window for assessing therapeutic efficacy and predicting long-term outcomes. A recent study validated the utility of longitudinal whole-blood transcriptomics for predicting outcomes in irradiated NHPs [81]. Pooling RNA biomarkers from Days 3 to 21 post-irradiation provided the highest predictive accuracy for survival in a rhesus macaques thoracic radiation model [81]. This specific timeframe overlaps with the BIO 300 Peak response phase identified in our study, supporting the clinical relevance of our temporal biomarker windows. Blood-based monitoring of the pathway signatures identified in this study, including adaptive immunity, phagocytosis, and metabolic reprogramming pathways could provide prognostic biomarkers of successful hematopoietic recovery. Second, the hemostatic signatures at Day 14 provide earlier indicators of bone marrow recovery, potentially enabling proactive clinical management decisions. Third, transcriptomic signals associated with neuroimmune pathways were detected in peripheral blood at Day 7; however, as these findings are derived from bulk blood RNA-seq, they reflect peripheral immune cell gene expression and cannot be interpreted as evidence of central nervous system involvement or neuroimmune-axis remodeling without tissue-specific validation.

The broader challenge of translating radiation countermeasures from animal models to clinical application remains a significant hurdle in the field. A recent comprehensive review of anti-radiation drug development over the past three decades identifies a persistent mechanistic knowledge gap as a primary barrier to clinical translation, noting that despite expansion of drug development into novel mechanisms (including DNA damage repair, anti-inflammatory strategies, and nano-delivery systems), overall clinical translation rates remain low, with only a limited set of approved agents (cytokines and growth factors) available for H-ARS [15]. The longitudinal transcriptomic signatures identified in this study may help address this gap by providing mechanistic molecular evidence of BIO 300’s multiphasic systemic activity. Day 14 hemostatic signatures and Day 21 immune reconstitution patterns identified here represent candidate pharmacodynamic endpoints that could inform dose optimization and efficacy assessment in future clinical investigations under the FDA Animal Rule framework, pending independent validation. Our study has limitations that warrant consideration. The NHP model, while representing the gold standard for radiation countermeasure development under the FDA Animal Rule requirements, may not fully recapitulate human radiation responses [58]. The small sample size, while consistent with ethical and logistical constraints of NHP radiation research and with previous studies in this model, limits power for detecting subtle transcriptional changes [26]. Because radiation-induced mortality reduced sample sizes, differential expression, temporal patterns, and pathway enrichments at these affected timepoints warrant careful interpretation. The absence of a BIO 300-treated, non-irradiated control group is an important design limitation that constrains mechanistic interpretation. BIO 300’s active ingredient genistein has known baseline pharmacological activity, including selective estrogenic agonism at ERβ, anti-inflammatory modulation of NF-κB signaling, and immunomodulatory effects on circulating immune cells that may independently induce immune, metabolic, stress-related, or interferon-associated transcriptional changes in the absence of radiation. Consequently, the current design cannot fully distinguish drug-intrinsic transcriptional effects from those arising specifically from the interaction of BIO 300 with radiation-induced injury. This applies to the drug-specific gene category (6,214 unique genes), where transcriptional programs detected exclusively in BIO 300-treated animals cannot be unambiguously attributed to radioprotective mechanisms rather than to BIO 300’s baseline pharmacological activity. All drug-specific transcriptional responses should therefore be interpreted as candidates for radiation-context protective activity, pending confirmation in a study design that includes a non-irradiated drug-treated arm. This limits confidence in attributing the identified biomarker signatures solely to BIO 300 activity and warrants caution when extrapolating these biomarkers for human dose conversion. The transcriptomic findings have not been validated by quantitative RT-PCR, which will be performed in subsequent studies to confirm key gene expression changes and pathway responses. Additional limitations include the use of only male animals in the adult cohort and wide ranges in age and body weight, which were partly driven by the restricted availability and high cost of rhesus macaques following the COVID-19 pandemic [47]. Blood transcriptome reflects primarily circulating immune cell populations and may not capture tissue-specific responses in other radiosensitive organs. Future studies should integrate multi-omics approaches (proteomics and metabolomics) and tissue-specific analyses to provide comprehensive mechanistic insights.

## Conclusion

This longitudinal blood transcriptome study reveals that BIO 300-mediated radiation rescue operates through distinct biological phases spanning acute protection, immune reconstitution, and sustained recovery. During the acute injury phase (Days 7–10), BIO 300 preserves baseline cellular homeostasis, maintaining normal expression patterns across 745–865 genes despite severe radiation exposure. Protection remains robust during the immune reconstitution phase on Day 21 (681 genes). Late-phase sustained protection at Day 60 (558 genes) demonstrates BIO 300’s multiphasic efficacy against both acute radiation syndrome and delayed radiation effects.

A core set of 20 genes exhibiting sustained differential expression were identified as candidate exploratory biomarkers of BIO 300-mediated radioprotection at the transcriptional level. SOX2 and AKAP11 suppression is consistent with a shift in stemness-associated transcriptional programs that may reflect hematopoietic recovery, and upregulation of TIMD4, BTNL10, VNN2, and CLEC1A is consistent with transcriptional signatures associated with immune surveillance; however, these interpretations are based solely on RNA-seq temporal pattern classification and should be regarded as exploratory. Protein-level quantification, RT-PCR confirmation, and functional validation in independent cohorts are required before these candidates can be considered validated pharmacodynamic markers. BIO 300’s multi-modal mechanism operates through four distinct cellular response patterns. Protected genes maintain baseline functions such as p53 signaling and proteostasis. Reversed genes actively counteract radiation injury, for example by restoring antigen presentation. Drug-specific genes were associated with transcriptional programs including immune activation and erythropoiesis; however, as a non-irradiated BIO 300 control group was not included in this study, these responses cannot be fully distinguished from BIO 300’s baseline pharmacological activity and should be considered candidate radioprotective signatures pending further validation. Attenuated genes prevent excessive stress responses. The identification of temporally orchestrated pathway signatures supports development of potential blood-based biomarker panels for monitoring therapeutic efficacy. These include hemostatic recovery on Day 14, immunometabolic reconstitution on Day 21, and neuroimmune modulation on Day 7 (with a nominal signal at Day 38 warranting further validation). These findings support BIO 300 development as a prophylactic agent for acute radiation syndrome while highlighting the underappreciated role of neuroimmune interactions in systemic radiation responses, with direct translational potential for clinical dose optimization and patient monitoring strategies.

## Electronic supplementary material

Below is the link to the electronic supplementary material.

Supplementary material 1

Supplementary material 2

Supplementary material 3

Supplementary material 4

Supplementary material 5

Supplementary material 6

## Data Availability

The combined transcriptomics data have been deposited to the Dryad Digital Repository, and the published link is available: https://datadryad.org/dataset/doi:10.5061/dryad.vmcvdnd66.

## Declaration

### Disclaimer

The opinions or assertions contained herein are the private views of the authors and are not necessarily those of the Uniformed Services University of the Health Sciences, or the Department of War.
