# Supplementary figures and images for: Multiphasic blood transcriptomic signatures of radioprotection by BIO 300, a synthetic genistein nanosuspension, in a nonhuman primate model of acute radiation syndrome
Source: J Transl Med. 2026 Jun 30;24:835. doi: 10.1186/s12967-026-08485-4 (PMC13321660; doi:10.1186/s12967-026-08485-4)

# PCA: Treatment Effect

PC1 (27.4%) vs PC2 (14.7%)

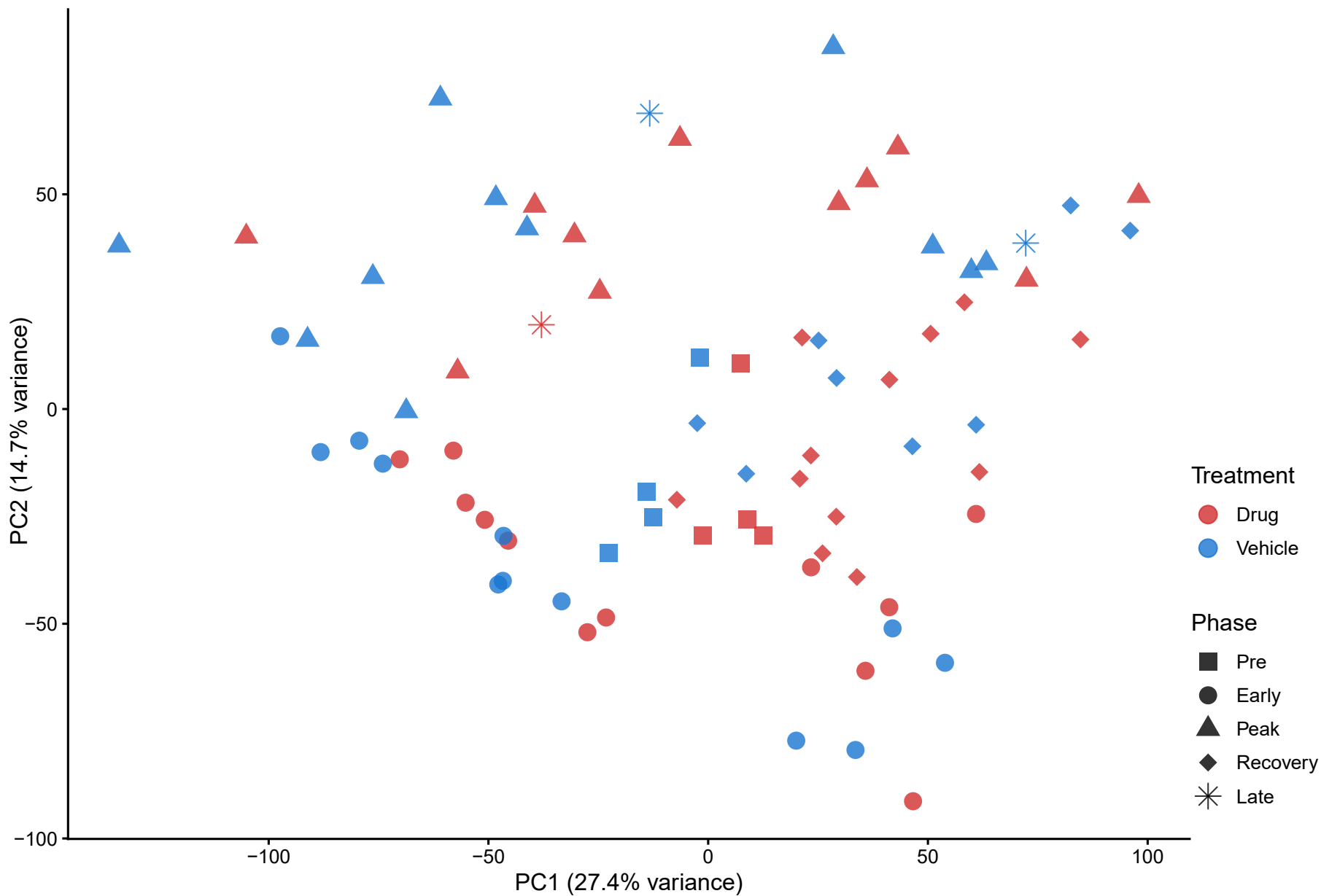

Supplement: Supplementary file 1 — Supplementary material 1 [file 12967_2026_8485_MOESM1_ESM.pdf]

# PCA: Temporal Trajectory

PC1 (27.4%) vs PC2 (14.7%)

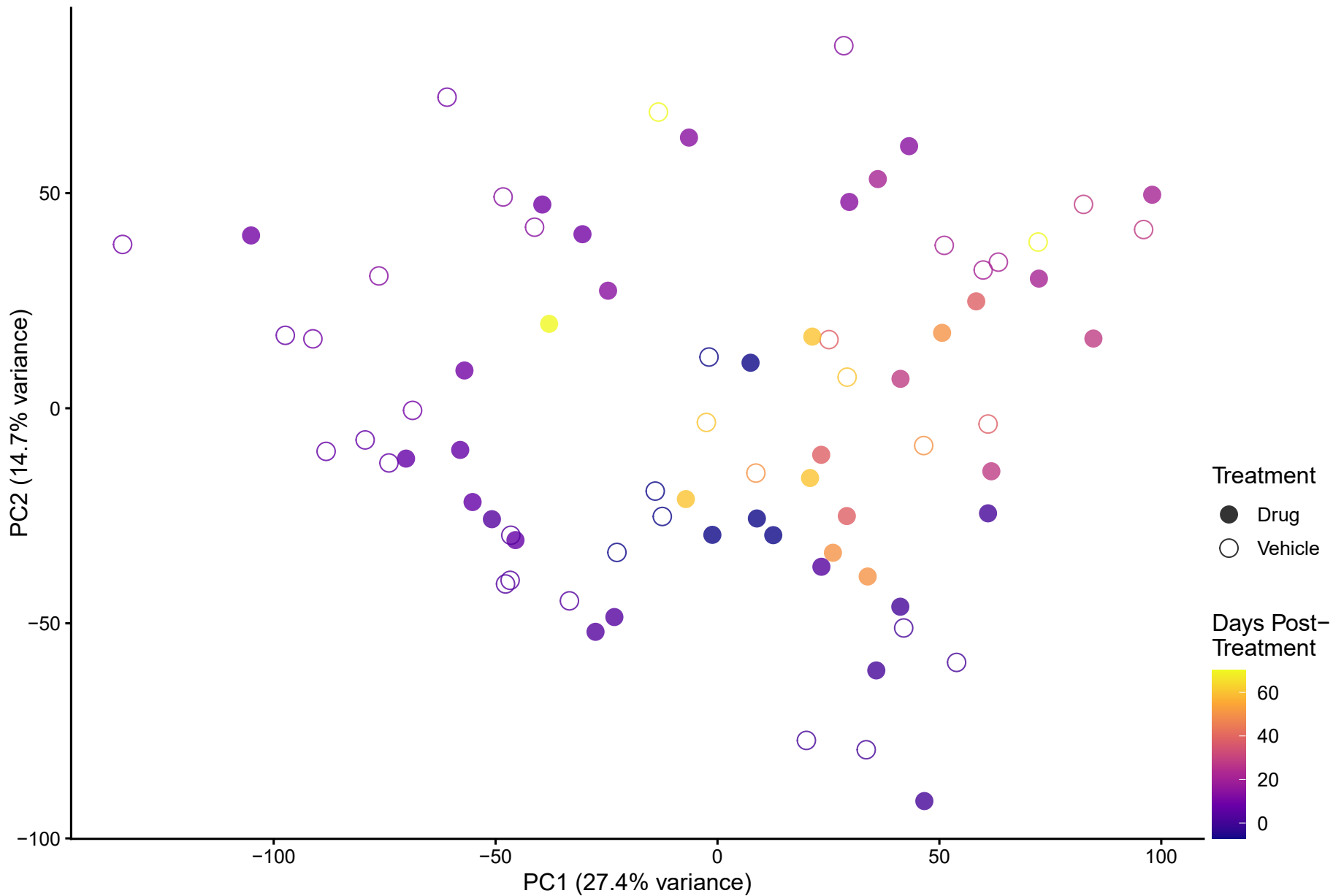

Supplement: Supplementary file 2 — Supplementary material 2 [file 12967_2026_8485_MOESM2_ESM.pdf]

Hierarchical Clustering of Samples (Ward's Method)

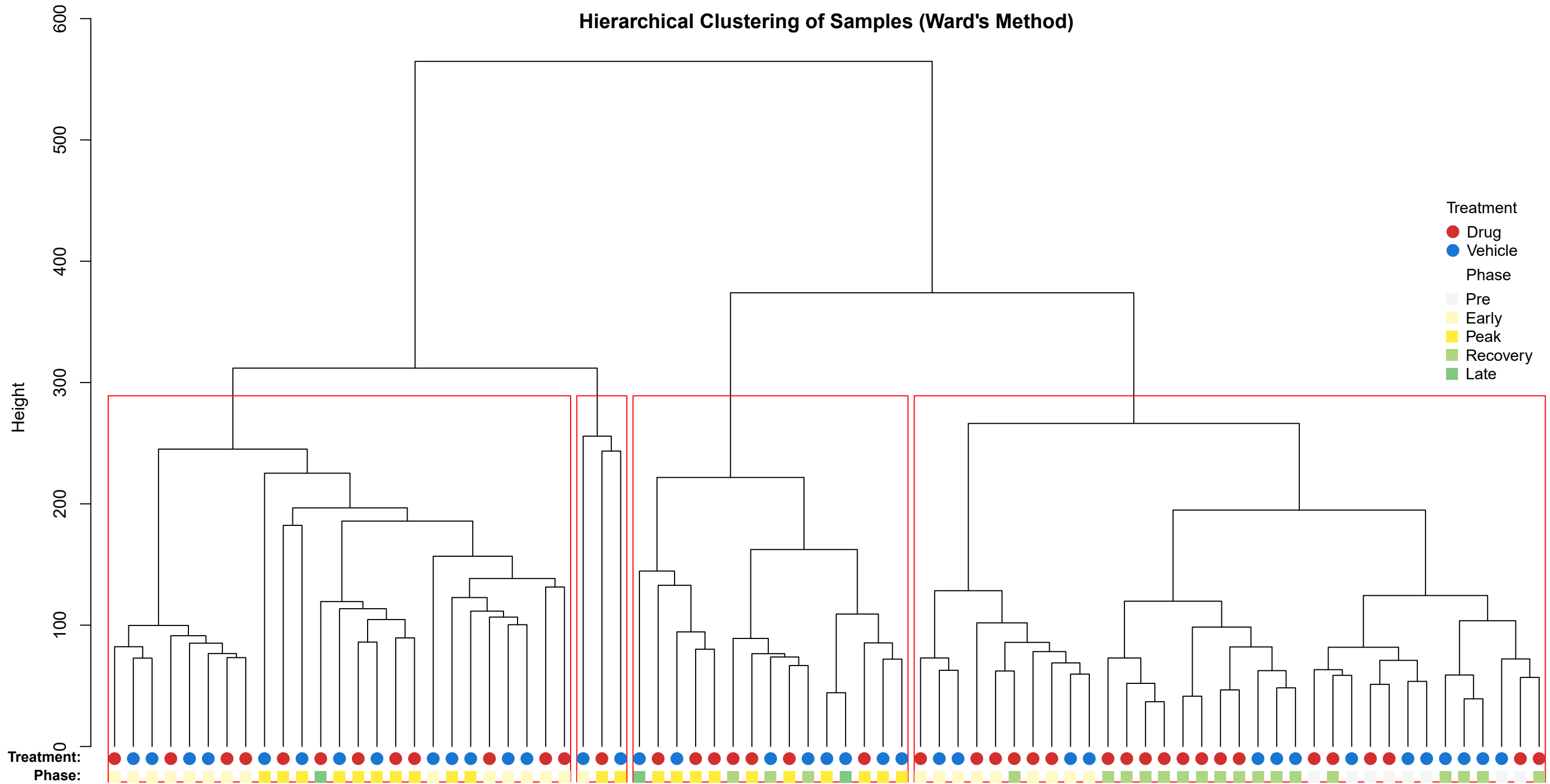

Supplement: Supplementary file 3 — Supplementary material 3 [file 12967_2026_8485_MOESM3_ESM.pdf]

# GO BP - Day21

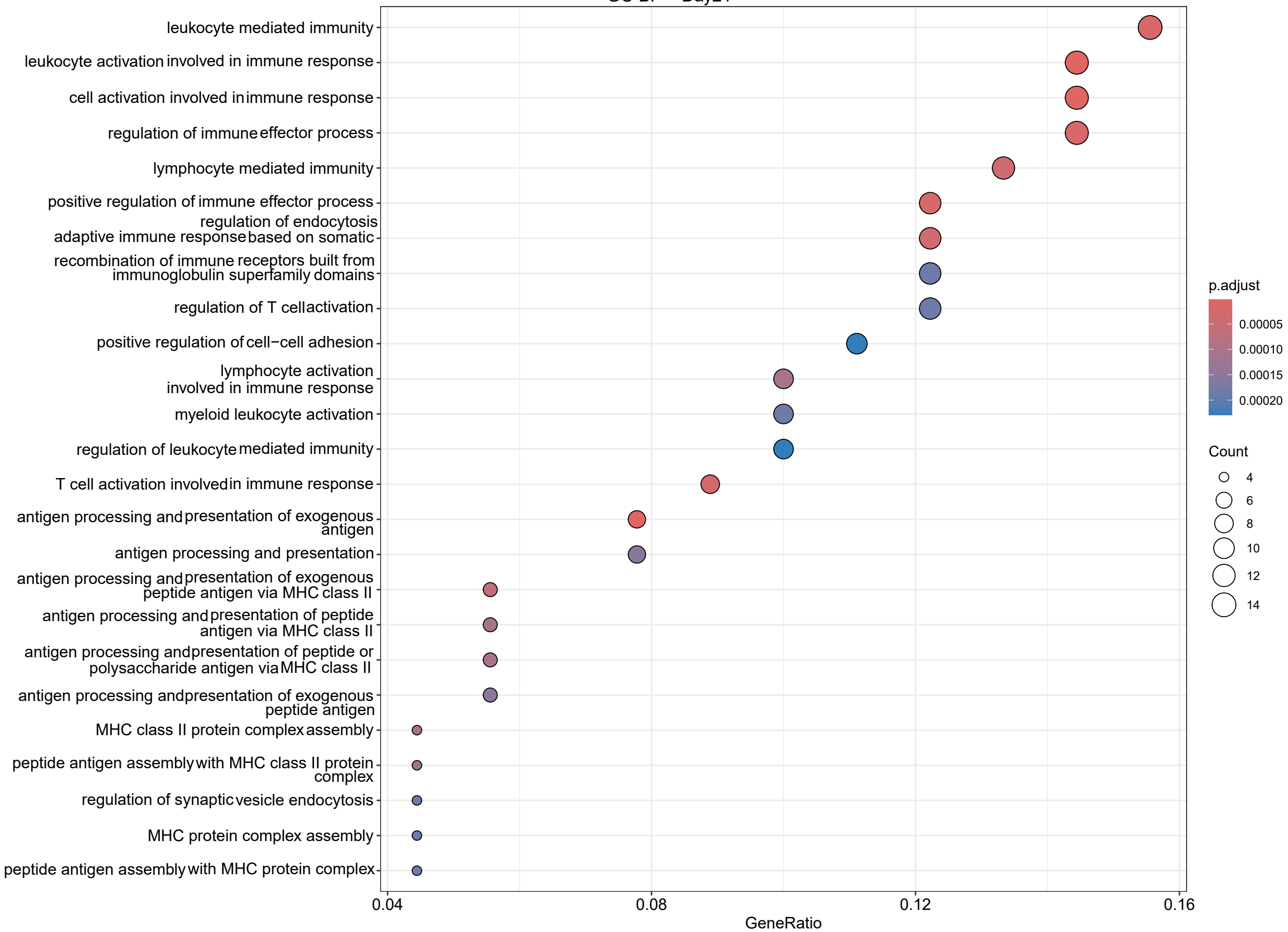

Supplement: Supplementary file 4 — Supplementary material 4 [file 12967_2026_8485_MOESM4_ESM.pdf]
