# Supplementary material for: Multiphasic blood transcriptomic signatures of radioprotection by BIO 300, a synthetic genistein nanosuspension, in a nonhuman primate model of acute radiation syndrome
Source: J Transl Med. 2026 Jun 30;24:835. doi: 10.1186/s12967-026-08485-4 (PMC13321660; doi:10.1186/s12967-026-08485-4)
Supplement: Supplementary file 6 — Supplementary material 6 [file 12967_2026_8485_MOESM6_ESM.docx]

**Supplementary Table 1A:** Vehicle Pre Treatment Day -7vs Day1 radiation response

**Supplementary Table 1B:** Vehicle Pre Treatment Day -7vs Day4 radiation response

**Supplementary Table 2A:** Drug Pre Treatment Day -7vs Day1 radiation response

**Supplementary Table 2B:** Drug Pre Treatment Day -7vs Day4 radiation response

**Supplementary Table 3:** Pattern classification of differentially expressed genes across all time points

**Supplementary Table 4:** Summary statistics of priority genes identified

**Supplementary Table 5:** Details of priority genes identified

**Supplementary Table 6:** GO-BP pathways at Day4

**Supplementary Table 7:** GO-BP pathways at Day 7

**Supplementary Table 8:** GO-BP pathways at Day 10

**Supplementary Table 9**: GO-BP pathways at Day 14

**Supplementary Table 10**: GO-BP pathways at Day 21

**Supplementary Table 11:** GO-BP pathways at Day 28

**Supplementary Table 12:** GO-BP pathways at Day 38

**Supplementary Table 13:** GO-BP pathways at Day 50

**Supplementary Table 14:** GO-BP pathways at Day 60

**Supplementary Table 15:** GO-BP pathways at Pre-Final

**Supplementary Table 16:** Reactome pathway enrichment Day4

**Supplementary Table 17:** Reactome pathway enrichment Day7

**Supplementary Table 18:** Reactome pathway enrichment Day10

**Supplementary Table 19:** Reactome pathway enrichment Day14

**Supplementary Table 20:** Reactome pathway enrichment Day21

**Supplementary Table 21:** Reactome pathway enrichment Day28

**Supplementary Table 22:** Reactome pathway enrichment Day38

**Supplementary Table 23:** Reactome pathway enrichment Day50

**Supplementary Table 24:** Reactome pathway enrichment Day60

**Supplementary Table 25:** Reactome pathway enrichment Pre-Final

**Supplementary Table 26:** KEGG Enrichment Day4

**Supplementary Table 27:** KEGG Enrichment Day7

**Supplementary Table 28:** KEGG Enrichment Day10

**Supplementary Table 29:** KEGG Enrichment Day14

**Supplementary Table 30:** KEGG Enrichment Day21

**Supplementary Table 31:** KEGG Enrichment Day28

**Supplementary Table 32:** KEGG Enrichment Day38

**Supplementary Table 33:** KEGG Enrichment Day50

**Supplementary Table 34:** KEGG Enrichment Day60

**Supplementary Table 35:** KEGG Enrichment Day Pre-Final

**Supplementary Table 36:** Time-Course Patterns of Gene Response to BIO 300

**Supplementary Table 37:** Gene response to BIO 300 at different time points

**SF1.** Principal component analysis (PCA) of RNA-seq expression profiles from BIO 300-treated (red) and vehicle-treated (blue) rhesus macaques across all post-irradiation time points, with point shape indicating experimental phase (Pre, Early, Peak, Recovery, Pre-Final). PC1 (27.4% variance) and PC2 (14.7% variance) are shown.

**SF2.** PCA of RNA-seq profiles as in SF1, with point color indicating time post-irradiation (dark purple = early, yellow = Day 60) and fill indicating treatment (filled = BIO 300, open = vehicle). The color gradient reveals a temporal trajectory across the PCA space, reflecting progressive transcriptomic changes over the post-irradiation time course.

**SF3.** Hierarchical clustering dendrogram (Ward's method) of all samples based on genome-wide RNA-seq expression profiles, with color bars indicating treatment (BIO 300 vs vehicle) and phase. Samples cluster predominantly by phase rather than treatment, consistent with radiation injury being a dominant driver of transcriptomic variation at 5.8 Gy.

**SF4.** Gene Ontology Biological Process (GO-BP) enrichment dot plot for differentially expressed genes at Day 21 post-irradiation (BIO 300 vs. vehicle). Dot size represents the number of enriched genes per term and dot color indicates adjusted p-value; immune activation and antigen presentation pathways show the strongest enrichment at Day 21.
